# An observational multi-center study on type 2 diabetes treatment prescribing pattern and patient adherence to treatment

**DOI:** 10.1038/s41598-023-50517-2

**Published:** 2023-12-27

**Authors:** Muhammad Daoud Butt, Siew Chin Ong, Azra Rafiq, Tooba Malik, Ahsan Sajjad, Nighat Batool, Anwaar Ul Hassan Chughtai, Muhammad Umar Wahab, Muhammad Abdullah, Zaheer-Ud-Din Babar

**Affiliations:** 1 Discipline of Social and Administrative Pharmacy, School of Pharmaceutical Sciences, Universiti Sains Malaysia, 11800 Penang, Malaysia; 2 Department of Pharmacy, Punjab University College of Pharmacy, Allama Iqbal Campus, University of the Punjab, Lahore, 54000 Pakistan; 3 Faculty of Biological Sciences, Quaid-i-Azam University, Islamabad, 45320 Pakistan; 4 Department of Public Health, Health Services Academy, Islamabad, 45320 Pakistan; 5 Ibn Sina Community Clinic South Wilcrest Drive, Houston, TX 77099 USA; 6 Faculty of Medicine and Community Health, University of Oslo, Oslo, Norway; 7 Middle East Technical University, Ankara, Turkey; 8 Umar Diabetes and Foot Care Centre and Umar Diabetes Foundation, Office 1, Executive Complex, G8 Markaz, Islamabad, 46000 Pakistan; 9 Pak-Austria Fachhochschule: Institute of Applied Sciences and Technology, Haripur, KPK Pakistan; 10 Department of Pharmacy, University of Huddersfield, Huddersfield, UK; 11 Department of Pharmacy, Riphah International University, Lahore, Pakistan

**Keywords:** Health care, Medical research

## Abstract

In 2021, the International Diabetes Federation (IDF) reported that the prevalence of diabetes in Pakistan was 9.6%, higher than the global average. However, adherence to treatment guidelines, e.g., American Diabetes Association and Pakistan Endocrine Society and prescription patterns for Oral anti-diabetes (OAD), is poorly understood in Pakistan. Therefore, this study aimed to examine the prescribing practices of anti-diabetic medications, an association of lifestyle modification with drugs prescribed, and their effectiveness in preserving ideal glycemic levels in diabetic patients undergoing treatment in tertiary care teaching hospitals in rural and urban Pakistan. In this cross-sectional study, data were collected from prescriptions of outpatient diabetic patients from different rural and urban tertiary care hospitals between October 2021 and February 2022. 388 participants were enrolled in the study for a detailed interview on prescription evaluation and glycemic control. The coinvestigators conducted an interview with the patient and used a pre-validated questionnaire to collect the data. The relationship between following treatment guidelines and clinical and demographic factors was found using chi-square tests for bivariate analyses. The study reported that out of 388, the mean ages of the patients were 48 ± 12.4, and the majority were female. It was observed that 60.1% and 66.5% have uncontrolled fasting and random blood glucose, respectively. The education level of the study participants was also below par to have a complete understanding of the medical condition and self-management therapy. Even though they were taking the right medications—an average prescription regimen included 5.08 medications—52.1% of the studied people had glycated haemoglobin (HbA1c) levels higher than the therapeutic threshold set by the International Diabetes Federation. In this modern era, it was observed that the prescribing trend was still focused on traditional therapeutic options Biguanides, sulfonylureas, and dipeptidyl peptidase-4 inhibitors were prescribed in 64.6% of the patients. A significant association was found between glycemic control and body mass index, adherence to lifestyle modifications, and the number of medications prescribed (p-value < 0.05). The study reveals that Pakistan's prescribing practices do not align with international and national guidelines, leading to a high prevalence of uncontrolled diabetes and widespread use of polypharmacy among patients. To address this issue, policymakers should prioritize establishing a comprehensive national diabetes action plan. Additionally, there is a pressing need to develop diabetes education and awareness programs emphasizing the importance of lifestyle modifications for effective diabetes management.

## Introduction

Diabetes mellitus, more called diabetes, is a metabolic disorder of multiple etiology characterized by chronic hyperglycemia with disturbances of carbohydrate, fat, and protein metabolism resulting from defects in insulin secretion, insulin action, or both^1^. Type 2 diabetes is the most common type of diabetes, accounting for over 90% of all diabetes worldwide. In type 2 diabetes, hyperglycemia is the result, initially, of the inability of the body’s cells to respond fully to insulin, a condition termed insulin resistance. According to data from the International Diabetes Federation (IDF) Atlas 10th edition, it was reported that 537 million people were living with diabetes worldwide. However, 74 Million new diabetics were included in the pool from the last estimate made in 2019. There was an overall 16% increase in the population of diabetes globally, and estimatedly, there will be a 46% (783 Million) increase globally by 2045^2–4^. According to the 10th Edition of the IDF Diabetes Atlas, released on December 6th, 2022, Pakistan is ranked 3rd globally with almost 33 Million diabetics. It has the highest national prevalence (26.7%) of diabetes globally, with one in four persons reporting having the disease^5^.

In a recent review study published by Ramzan et al., they comprehensively covered the pattern of anti-diabetes prescription^6^. It was reported that the global prescription pattern of anti-diabetic medications varies in different regions of the world^7–9^. Metformin is the first-line oral anti-diabetic agent recommended in most guidelines published by the American Association of Clinical Endocrinology and the International Diabetes Federation^10,11^. In the developed nations, those following the American Diabetes Association and European Association for the Study of Diabetes guidelines tend to have higher prescription levels of glucagon-like peptide-1 GLP-1 and sodium-glucose-linked transporter-2 SGLT-2^6,12,13^. In accumulation to metformin, there were many different combinations of sulfonylureas, dipeptidyl peptidase 4 DPP-4 inhibitors, sodium-glucose linked transporter-2 (SGLT2) inhibitors, and receptor agonists similar to glucagon-like peptide-1 (GLP-1) are commonly prescribed based on the clinical characteristics, co-morbidities, and treatment goals of the individual patient^14,15^. Various studies reported evolving trends in diabetes medication across European countries. Sulfonylurea use declined, notably in France, which dropped from 35 to 29% between 2001 and 2003 and across Europe between 2008 and 2012. In Spain and Germany, sulfonylurea prescriptions were around 17% and 12%, respectively, with the UK seeing a reduction from 10 to 6% between 2006 and 2010 and even lower for first-line use. Conversely, metformin saw increased usage in France, Germany, and the UK, reaching 91% as a first-line treatment in the UK in 2013. Insulin use varied, increasing from 1.71 to 2.27% in France, while the UK maintained consistent usage. Thiazolidinediones initially surged but declined in most countries, and Dipeptidyl peptidase-4 inhibitors gained popularity, particularly in France and the UK. Glucagon-like peptide-1 receptor agonists showed mixed trends^6,12,16–18^.

Prescription patterns define the type and character of medication use and adherence to local, state, or federal regulations, such as standardized prescribing practices, the use of medications from the essential drug list, and the use of generic medications^19,20^. Adherence and the prevention of disease are both advantages of appropriate prescribing. An irrational prescribing pattern develops when the wrong anti-hyperglycemic drug dosage, frequency, or duration is administered^21,22^. Due to inadequate therapy and unpleasant drug reactions, improper drug administration may also increase patient costs. Loss of confidence between the patient and the practitioner may result from a lack of progress and adverse drug reactions^23^.

The management of diabetes in Pakistan is challenging due to limited resources, poor access to health care, and a lack of awareness among the general population. Patients with diabetes in Pakistan are managed with a combination of lifestyle modifications and pharmacological interventions^24^. However, adherence to treatment guidelines and the use of oral antihyperglycemic agents in Pakistan needs to be clarified. Previous studies reported that metformin, sulfonylureas (SU), and dipeptidyl peptidase four inhibitors (DPP-4) are Pakistan's most commonly prescribed oral antihyperglycemic agents. There is a significant variation in the prescription patterns in different regions of the country^24,25^. There is a need for further research to evaluate prescribing trends and practices in diabetes management in Pakistan to ensure that patients receive optimal care^26,27^.

According to the United Kingdom Prospective Diabetes Study, intense blood glucose control with sulfonylureas or insulin significantly reduced the risk of microvascular complications^28,29^. A significant disparity was observed in Pakistan, with multiple research documenting suboptimal prescribing practises. Various studies conducted in different provinces have documented a notable deficiency in medication adherence within the Punjab district. Specifically, it has been seen that a significant proportion of patients, approximately 68%, adhere solely to the first-line recommended therapy, namely Metformin, for the purpose of managing their diabetes. According to a recent survey, persons aged 65 and above with diabetes were prescribed an average of 8.3 medicines^30^. According to the available data, a notable proportion of patients, specifically 18.7%, were found to have been prescribed with inaccurate dose or inappropriate dosage forms^31,32^.

The prescribing patterns of antidiabetic medications in Pakistan represent a pressing concern due to the high prevalence of diabetes within the Pakistani population. Prioritizing the use of optimal therapies is essential for effective blood sugar management and the prevention of potential complications. Nevertheless, there is a clear need for in-depth research into the specific trends in the prescription of antidiabetic drugs in Pakistan, underscoring the necessity for further investigation^33^. Varied regional patterns exist in oral antihyperglycemic agent prescriptions in the country. Fast and long-acting insulin analogs saw increased use, while natural insulin prescriptions decreased significantly (p < 0.001). Metformin, incretin modulators, and fixed oral combinations were prescribed more frequently (p < 0.001). Human insulin was preferred for female and elderly patients^34,35^. Older patients were less likely to be prescribed metformin and more likely to be prescribed sulphonylureas. There were studies that reported that adding sulphonylureas to the therapy may increase the risk of mortality and risk of hypogycemia^36^. In the non-means-tested scheme, socioeconomic distinctions were observed in the increased prescribing of newer, more expensive anti-diabetic drugs. Both insulin and oral anti-diabetic medications were prescribed in a manner that varied by region^37^. In addition, there needs to be more adherence to treatment guidelines, and patients may receive suboptimal therapy. Therefore, it is necessary to evaluate the prescribing patterns of anti-diabetic medications in Pakistan to identify areas for improvement and ensure that patients receive the best possible care^38^.

Therefore, this study aimed to examine the prescribing practices of anti-diabetic medications, an association of lifestyle modification with medications prescribed, and their effectiveness in preserving ideal glycemic levels in diabetic patients undergoing treatment in tertiary care teaching hospitals in rural and urban Pakistan.

## Methodology

### Study design

This cross-sectional observational study was conducted in six different tertiary care hospitals from 3 major cities of Pakistan. The study population includes patients with type 2 diabetes mellitus who received care at selected healthcare facilities during the study period (October 2021 and February 2022).

### Inclusion criteria

1. Participants had a confirmed diagnosis of type 2 diabetes mellitus based on established diagnostic criteria such as fasting plasma glucose levels, oral glucose tolerance test, or glycated hemoglobin (HbA1c) levels.
2. Participants were 18 years or older and were citizens or residents of Pakistan.
3. Written Informed Consent: participants could provide written informed consent or have a legally authorized representative provide support.
4. Participants were able to provide written informed consent.
5. Participants were on a stable oral antihyperglycemic drug regimen for at least three months before enrollment.

### Exclusion criteria

1. Participants diagnosed with type 1 diabetes mellitus were excluded from the study as the focus was on type 2 diabetes mellitus.
2. Pregnant or lactating females were excluded from the study due to potential risks associated with medication use during these conditions.
3. Participants with severe renal or hepatic impairment, as determined by clinical assessment or laboratory tests, were excluded due to the potential impact on the metabolism and excretion of oral antihyperglycemic drugs.
4. Participants with a history of diabetic ketoacidosis were excluded due to the potential need for specialized treatment and management.

### Ethical approval and consent to participate

The study received ethical approval from the Human Research Ethical Committee of Universiti Sains Malaysia (USM/JEPeM/22090589), the Bio-Ethics Committee (BEC) of Bahauddin Zakariya University, Multan (ACAD/EXT/01/2022), and the Ethical Review Board of Shaheed Zulfkar Ali Bhutto Medical University (1-3/2019/ERB/SZABMU). It was conducted in multiple healthcare facilities throughout Pakistan, both public and private, including Pakistan Institute of Medical Sciences, Capital Development Authority (CDA) hospital, Islamabad, District Health Quarter Hospital, Rawalpindi, City International Hospital Quetta, Royal Healthcare Multan, and Medicare Hospital Rawalpindi. Ethical approval was obtained from the relevant institutional review boards of all participating hospitals, and written informed consent was obtained from study participants with confidentiality maintained throughout the study. The study was conducted per the Declaration of Helsinki and reported according to “The Strengthening the Reporting of Observational Studies in Epidemiology (STROBE)” guidelines^39,40^.

### Sampling size

The Rao Soft calculator calculated 384 mandatory sample sizes based on 33% prevalence, 5% margin error, and 95% confidence interval^41^. The sample size was determined based on the total number of patients with type 2 diabetes mellitus who received care in the selected healthcare facilities during the study period. For this purpose, 450 patients were interviewed, and 388 participants met the study criteria.

$${\text{N}} = \left[ {{\text{Z}}^{{2}} *{\text{p}}*\left( {{1}{-}{\text{p}}} \right)} \right]/\left( {{\text{E}}^{{2}} } \right)$$

where: n is the required sample size, Z is the Z-value corresponding to the desired level of confidence (e.g., 1.96 for a 95% confidence level), p is the estimated prevalence or proportion in the population, E is the desired margin of error or precision.

### Data collection

Data were collected by reviewing the medical records of the patients. A predesigned data collection form was used to record the following variables: patient demographics, clinical characteristics, prescription of antihyperglycemic drugs, dosing, and adherence to treatment guidelines. The prescription pattern was analyzed, and compliance with treatment guidelines was determined by comparing it to the ADA’s 2021 recommendations. Several parameters, including Hb1Ac and adherence to therapy, were considered when evaluating prescription adherence to ADA guidelines.

To access the medication adherence level of the study participants, urdu validated version of the Drug Attitude Inventory 10 Items (DAI-10).

**Figure 1:**
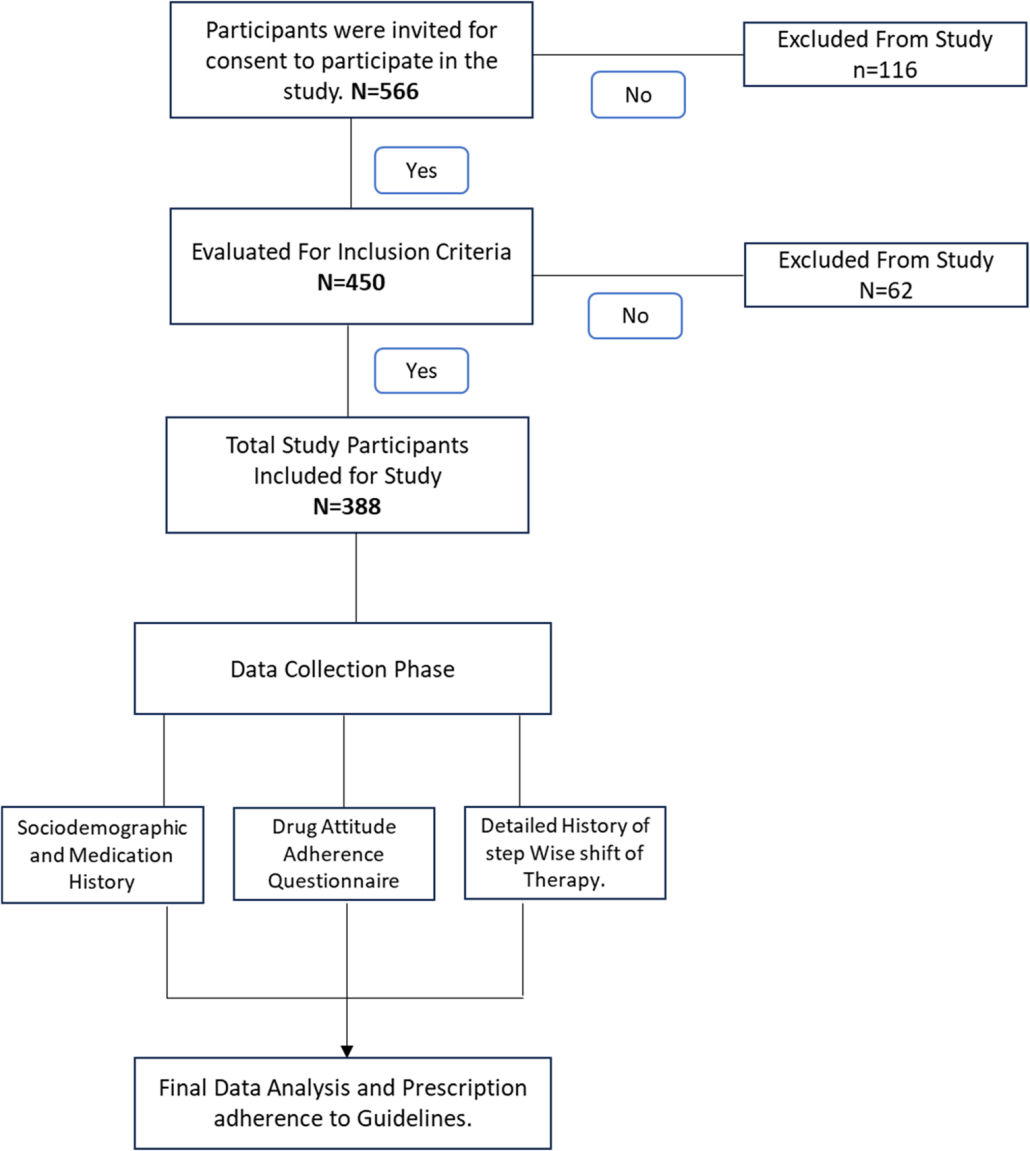
Flow of study participants recruitment and assessment.

The flow of study is as follows shown in Fig. 1.

### Study variables

The study variables include Age, sex, duration of diabetes, body mass index (BMI), co-morbidities, prescription of antihyperglycemic medications, and dose, including the laboratory values of the patients, i.e., HbA1c, fasting blood glucose, random blood glucose, and adherence to treatment guidelines.

### Data analysis

Data were analyzed using descriptive statistics, including mean, median, standard deviation, and frequency distributions. A chi-square test was applied to assess the association between prescribed medication and adherence to the recommended guidelines. Statistical analysis was performed using Microsoft Excel 2016 and SPSS version 22.

## Results

The study results demonstrate that there were 388 patients, including men (180) and women (208). Patients were divided into five age groups. There were 152 (39%) between 41 and 50 years and 129 (33.2%) between 51 and 60 years. 233 (60.1%) of 388 patients had Fasting Blood Glucose (FBG) > 126 mg/dl, and 258 (66.5%) had Random Blood Glucose (RBG) > 200 mg/dl.

The study has yielded crucial insights into the characteristics and distribution of type 2 diabetes patients. Of particular interest is the fact that 39.0% of diabetes cases were found in the age group of 41–50 years. In comparison, only 1.5% were seen in individuals aged 28–30 years, indicating a significant age-related trend in diabetes prevalence. Additionally, the study found that more females (53.6%) were affected by diabetes than males (46.4%), which is an important observation. Education-wise, over half of the diabetic patients (57.7%) had only a primary level of education, highlighting a significant educational distribution among the diabetic population. Occupation-wise, housewives constituted a substantial proportion of patients (50.8%), indicating the important role of this occupation in diabetes prevalence. Government employees (23.2%) and businessmen (14.9%) also showed significant representation within the diabetic cohort. The study also revealed that the most prevalent co-morbidity was a combination of diabetes and hypertension, affecting 32.5% of patients, providing crucial insights into the health profile of diabetic individuals. Lastly, family history was found to be an essential factor, with the majority (71.6%) of patients having a positive family history of diabetes (Table 1).

**Table 1:** Demographic characteristics of study participants (n = 388).

Parameters	Number (mean ± SD)	Percent (%)
Age (years)		
(range 28–70)	388 (48 ± 12.4)	
Age groups (years)		
28–30	6	1.5
31–40	63	16.2
41–50	152	39.2
51–60	129	33.2
61–70	38	9.8
Gender		
Male	180	46.4
Female	208	53.6
Education		
Primary	223	57.5
Higher secondary	120	30.9
Secondary	45	11.6
Occupation		
None	14	3.6
House wife	197	50.8
Govt. job	90	23.2
Private jobs	29	7.5
Business	58	14.9
Co-morbidities		
None	228	58.7
Hypertension	126	32.5
Osteoarthritis	24	6.2
Ischemic heart disease	4	1.0
Other diseases	6	1.5
Family history of DM		
Yes	278	71.6
No	110	28.4

In the analysis of laboratory parameters, it's worth noting that most patients (60.1%) had a fasting blood glucose level above 126 mg/dl, while 66.5% had a random blood glucose level exceeding 200 mg/dl. Regarding HbA1c levels, more than half of the patients (52.1%) had levels above 7.0. These results are significant as they highlight the prevalence of elevated blood glucose and HbA1c levels among the studied population, underscoring the need for effective diabetes management strategies.

A study of medication usage was observed in patients with Type 2 diabetes in a total of 388 prescriptions. The mean number of antihyperglycemic drugs per prescription was found to be 5.08, with a range of 1 to 3 drugs per prescription. The percentage of antihyperglycemic drugs prescribed was calculated as follows: 13.7% for Biguanides, 1% for DPP4i, 18.3% for Biguanides plus DPP4i, 5.7% for Sulfonylurea, 14.4% for Human Insulin, 3.9% for Sodium-Glucose Linked Transporter 2 (SGLT-2) plus Modern Insulin (insulin analog), 26% for Biguanides plus Sulfonylurea and DPP4i, 13.1% for Biguanides plus DPP4Ii and Insulin, 3.4% for Biguanides plus Sodium-Glucose Linked Transporter 2 (SGLT-2), and only 0.5% for SGLT-2 and DPP4i (Table 2).

**Table 2:** Clinical parameters of study participants.

Parameters	Number (Mean ± SD)	Percent (%)
BMI (kg/m^2^)		
Normal (18.5–24.9)	38 (22.3 ± 1.7)	9.8
Overweight (25–30)	219 (27.9 ± 1.1)	56.4
Obese (above 30)	131 (33.5 ± 1.2)	33.8
Life style		
Active	246	63.4
Sedentary	142	36.6
Blood sugar (fasting)		
< 100	40 (94.5 ± 7.9)	10.3
101–125 mg/dl	115 (119.4 ± 12.1)	29.6
> 126 mg/dl	233 (129 ± 8.8)	60.1
Blood sugar (random)		
< 140 mg/dl	32 (128.7 ± 4.9)	8.2
141–199 mg/dl	98 (161.5 ± 12.6)	25.3
> 200 mg/dl	258 (228.1 ± 27.1)	66.5
HbA1c (%)		
< 5.7	2	0.5
5.7–6.4	17	4.4
6.5–7.0	167	43
7.1 and above	202	52.1

**Figure 2:**
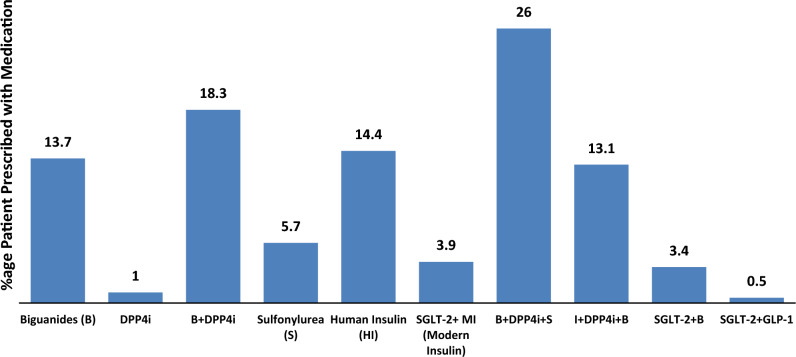
Percentage of medication use in the study sample. *DPP4I* dipeptidyl peptidase 4 inhibitors, *SU’s* sulphonylureas, *HI* human insulin, *MI* modern insulin/insulin analogue, *SGLT-2* sodium-glucose-linked transporter-2.

Further analysis of medication usage (Fig. 2) revealed that the majority of patients with Type 2 diabetes received triple therapy (26%), while 34.7% received monotherapy.

These findings suggest that healthcare providers use a combination of medications to effectively manage the condition, the most common combination being Biguanides, Sulfonylurea and DPP4i. These results have important implications for the management of Type 2 diabetes and highlight the need for individualized treatment plans to achieve optimal results.

The study on diabetes treatment combinations revealed that patients were administered monotherapy, dual therapy, and triple therapy. The utilization of Metformin, Sulphonylureas, and DPP-4 inhibitors has witnessed a notable rise in their application for the management of diabetes. The study revealed that a total of 50.6% of patients were treated solely with metformin, resulting in an average HbA1c reduction of 6.28%. A total of 21% of patients utilized sulphonylureas as a monotherapy, which led to a mean reduction in HbA1c levels of 6.86 units. Metformin and Sulphonylureas were administered to 48.3% of the patient cohort, resulting in a mean HbA1c value of 6.73. Nevertheless, a notable proportion of 53.1% of participants made a transition from Sulphonylureas to Metformin, which subsequently led to an average HbA1c level of 7.60. It is noteworthy that a significant proportion of patients, specifically 95.9%, were administered a combination of Metformin and DPP-4 inhibitors.

This treatment approach resulted in a mean HbA1c level of 7.87. The combination of Metformin with SGLT-2 inhibitors or GLP-1 agonists exhibited the highest level of glycated hemoglobin (HbA1c) at 10.33. The findings of the study indicate that the combination of Metformin-Sulphonylureas-DPP-4 is frequently employed as an effective treatment for diabetes. In contrast, the efficacy of other combinations in controlling HbA1c levels exhibited considerable variability as indiacated in the Table 3.

**Table 3:** Association of medication combination prescribed in correlation with the HbA1c levels.

Medications combinations	HbA1c %	
	Number (%) of patients corresponding to HbA1c value	Mean (95% CI)
Metformin monotherapy	50.6 (13.7)	6.28 (6.26, 6.29)
Sulphonylureas monotherapy	21.0 (5.7)	6.86 (6.80, 6.92)
Metformin plus sulphonylureas dual therapy	48.3 (13.1)	6.73 (6.70, 6.76)
Sulphonylureas plus metformin dual therapy	53.1 (14.4)	7.60 (7.54, 7.67)
Metformin to DPP-4 inhibitor dual therapy	95.9 (26)	7.87 (7.81, 7.92)
Metformin to sulphonylureas to SGLT-2 triple therapy	26.9 (7.3)	8.79 (8.73, 8.85)
Metformin to DPP-4 inhibitors to insulin triple therapy	67.5 (18.3)	8.38 (8.19, 8.58)
Metformin to SGLT-2 to GLP-1 agonist triple therapy	5.5 (1.5)	10.33 (10.09, 10.36)

**Figure 3:**
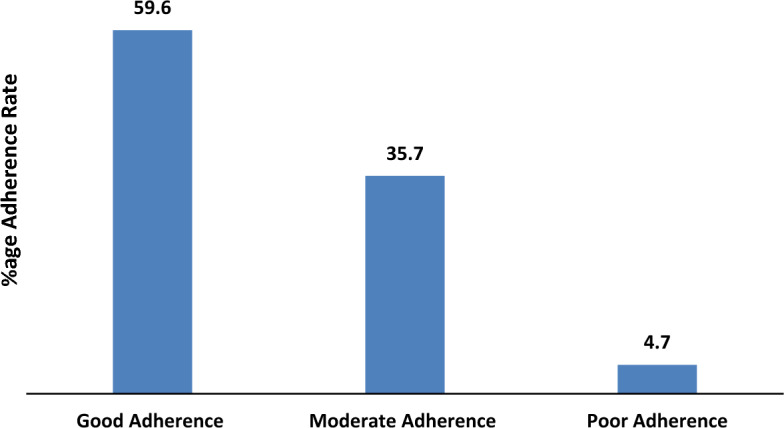
Level of treatment adherence observed during the study.

The data on treatment adherence reveals that 59.6% of patients demonstrated good adherence to their prescribed treatments, indicating a strong commitment to managing their health. Around 35.7% displayed moderate adherence, suggesting room for improvement, while 4.7% exhibited poor adherence as evident by Fig. 3.

To evaluate the association among sociodemographic characteristics, the chi-square test was applied, as represented in Table 4. The result of the association suggests that there was no significant association between glycemic levels and characteristics like gender, Age, and duration of treatment. However, a significant association was observed between patients with higher body mass index (BMI) (p-value < 0.001). It can be seen that there was a higher count of patients with uncontrolled glycemic levels falling into the overweight and obese category. Another important factor to compare among the wild study population is that the majority of them were on combination therapy, and despite using various oral antidiabetics, they are still unable to control their blood glucose levels (p-value < 0.05). In contrast, it was widely observed in the study population that those individuals who tend to follow the guidelines for lifestyle modifications given by the treating physician or diabetes educators have better glycemic control (p-value < 0.001). Overall, patients with better adherence to medical therapy and lifestyle modifications tend to have significantly better glycemic control.

**Table 4:** Association between sociodemographics of diabetic patients and glycemic control.

Characteristics	Glycemic control, n (%)		N	p-value
	Control	Uncontrolled		
Type 2 diabetes	186 (47.9)	202 (52.1)	388	
Age (years)				
< 40	9 (4.8)	60 (29.7)	69	0.711
40–60	154 (82.7)	127 (62.8)	281	
60–70	23 (12.3)	15 (7.4)	38	
Gender				
Males	81 (43.5)	99 (49.0)	180	0.12
Females	105 (56.5)	103 (51)	208	
BMI (kg/m^2^)				
Normal (18.5–24.9)	19 (10.2)	19 (9.4)	38	< 0.001*
Overweight (25–30)	86 (46.2)	133 (50.9)	219	
Obese (above 30)	81 (43.5)	50 (24.7)	131	
Duration of treatment (years)				
< 5	70 (37.6)	105 (51.9)	175	0.42
5–10	62 (33.3)	89 (44.0)	151	
> 10	27 (14.5)	35 (17.3)	62	
Type of anti-diabetic therapy				
Monotherapy	80 (43.0)	55 (27.7)	135	< 0.05*
Combination therapy	106 (57.0)	147 (72.7)	253	
Anti-diabetic therapy				
With lifestyle modifications	156 (83.8)	90 (44.5)	246	< 0.001*
Without lifestyle modifications	30 (16.2)	112 (55.5)	142	

## Discussion

The present study aimed to analyze the prescription patterns for oral antihyperglycemic drugs (OADs) and the adherence to treatment guidelines in patients with type 2 diabetes mellitus in Pakistan. The study findings revealed that the mean number of OADs prescribed per prescription was 5.08, with the most common combination being Biguanides, Sulfonylurea, and DPP4i. Type 2 diabetes mellitus is a chronic metabolic disorder resulting from insulin action and secretion defects. Type 2 diabetes mellitus has reached epidemic proportions worldwide; it has been studied that more than 220 million people had the disease in 2010^42^. A study shows that 336 million people have diabetes mellitus, and the number of diabetic patients will be 552 million by 2030^5^.

These results have important implications for managing type 2 diabetes in Pakistan. The high prevalence of poor glycemic control indicates that healthcare providers must reevaluate their treatment strategies and ensure that patients receive individualized treatment plans based on their medical history, lifestyle, and other co-morbidities. Additionally, healthcare providers should regularly monitor their patient's glycemic control to adjust treatment plans to achieve optimal outcomes^43^.

According to the Pakistan Endocrine Society (PES) guidelines 2021 (Appendix A), biguanides are first-line agents in type 2 diabetes. When glucose control targets are not achieved, add SGLT-2. If metformin is not used first-line, add a dipeptidyl peptidase-4 inhibitor (DPP-4), or a thiazolidinedione must be initiated in the patient. Moreover it was also recommended that is patient is having choronic obesity GLP-1 agonist must be initated. A rapid-acting insulin secretagogue is an alternative to sulfonylureas^44^.

Previous research has investigated the patterns of prescription for antidiabetic drugs within the Diabetes Registry Tyrol for the period spanning from 2012 to 2018. Significantly, Sodium/Glucose Cotransporter 2 inhibitors (SGLT-2i) exhibited a noteworthy rise in prescription rates (p < 0.001), along with metformin (p = 0.002), gliptins (p = 0.013), and glucagon-like peptide-1 agonists (GLP-1a) (p = 0.017). In contrast, there was a notable decrease in the number of prescriptions for sulfonylurea medications (p < 0.001). Metformin has been identified as the antidiabetic drug with the highest prescription rate, accounting for 51.3% of all prescriptions. Following metformin, insulin/analogs were the second most commonly prescribed drug at a rate of 34.6%. Gliptins were prescribed at a rate of 28.2%, while SGLT-2i, sulfonylurea, glitazones, GLP-1a, and glucosidase inhibitors were prescribed at rates of 11.7%, 9.1%, 3.7%, 2.8%, and 0.4% respectively. Significantly, the observed changes in prescription patterns remained consistent over the study, occurring prior to disseminating international and national guidelines. This suggests that physicians can adjust their practices based on developing clinical data, even before formal revisions to established standards^45–47^.

A comparison of the present study with previous studies conducted in Pakistan revealed some similarities and differences. For example, a study in Karachi reported that the most commonly prescribed OADs were Biguanides and Sulfonylureas, which is consistent with the present study. However, the study also reported that DPP4i was the most frequently prescribed class of OAD, which is in contrast to the present study^27^.

Furthermore, a study conducted in Lahore reported that 63% of the patients had poor glycemic control, similar to the present study's findings. However, the study also identified that Age, sex, and duration of treatment significantly affected glycemic control, in contrast to the current study's findings^48^.

Willey et al. study has shown that monotherapy can provide adequate glucose control. However, our investigation discovered a statistically significant relationship between glycemic control, monotherapy, and combination treatment. We found a statistically significant correlation between glycemic control, BMI, anti-diabetic medicine therapy, and lifestyle change^49–51^.

Therefore, even with optimal therapy with anti-diabetic medications, lifestyle change combined with anti-diabetic drug treatment can improve glycemic control in individuals with Type 2 diabetes.

The results of this study are consistent with previous research conducted in South Asia, which has also reported that metformin is the most commonly prescribed drug for diabetes mellitus type 2. For example, a study conducted in India by Tripathi et al. found that metformin was the most frequently prescribed drug, followed by sulfonylureas and DPP-4 inhibitors^52^. Another study conducted in Bangladesh by Akter et al. reported similar findings, with metformin being the most commonly prescribed drug, followed by sulfonylureas and meglitinides^53^.

However, the present study revealed a lower adherence rate to treatment guidelines than previous research conducted in Pakistan. A survey by Tabassum et al. reported that only 41.6% of total prescribed medications are in recommendation with the guidelines suggested by the American Diabetes Association^30^. Similarly, a study conducted in Lahore reported high adherence to treatment guidelines, with 80.5% of patients receiving the recommended medications^54^.

Our study's lower adherence rate to treatment guidelines could be attributed to several factors, including a need for more awareness among healthcare professionals about the latest treatment guidelines, poor patient education, and limited medication access. These findings highlight the need for healthcare professionals in Pakistan to be trained in the latest treatment guidelines and make more significant efforts to improve patient education and medication access.

The study has not considered other factors that could affect prescription patterns and adherence to treatment guidelines, such as the socioeconomic status of patients, access to healthcare, and cultural beliefs about diabetes management. These factors could affect the generalizability and applicability of the study findings.

## Conclusion

In conclusion, our study sheds light on imperative considerations for diabetes management in Pakistan. The findings underscore the critical necessity of tailoring treatment approaches to individual patients, considering their unique medical histories and lifestyles to achieve optimal glycemic control. The healthcare landscape must evolve to meet these demands, necessitating the implementation of updated training for healthcare providers, augmented patient education efforts, and enhanced medication accessibility.

As the prevalence of diabetes continues to rise in Pakistan, urgent systemic interventions are required. A comprehensive national action plan is essential, focusing on multifaceted strategies such as heightened public awareness, promotion of healthy lifestyle practices, and improved infrastructure for diabetes care and treatment access. Our study contributes substantively to the discourse on diabetes management in Pakistan, providing a foundation for evidence-based practices and policy initiatives.

Continued research is pivotal for a nuanced understanding of the complex factors influencing prescription patterns and guideline adherence in the Pakistani context. This ongoing inquiry will not only deepen our comprehension of diabetes management challenges but also inform future policies and practices, facilitating effective responses to the evolving health landscape. In essence, our research underscores the pressing need for tailored, informed, and dynamic approaches to diabetes care in Pakistan to address the increasing burden of this prevalent health concern comprehensively.

## Limitation

This study has limitations, focusing narrowly on physicians' antidiabetic prescribing practices while neglecting patient adherence, comorbidity management, and lifestyle adjustments crucial to diabetes care. Limited to established diabetic patients with year-long follow-ups, it misses early-stage insights. The exclusive reliance on quantitative data excludes nuanced perspectives from qualitative methods. Comparative analyses across regions and healthcare systems in Pakistan are lacking. Intervention studies to assess initiatives like educational programs are absent. To advance diabetes care research in Pakistan, future studies should embrace a longitudinal approach, integrate qualitative methods, conduct cross-regional comparisons, and include interventions for a more comprehensive understanding.

## Future recommendation

To advance type 2 diabetes management in Pakistan, crucial research avenues are outlined. Longitudinal studies over extended periods offer insights into prescribing patterns and adherence trends. Integrating qualitative methods, like interviews, enriches understanding. Regional comparisons within Pakistan illuminate prescribing variations, guiding targeted interventions. Intervention studies, focusing on education and communication strategies, optimize prescribing practices. Evaluating health system dynamics, technology's role, collaboration, and long-term cost-effectiveness refines diabetes care. Addressing these imperatives elevates knowledge and enhances type 2 diabetes management in Pakistan.

### Supplementary Information

Supplementary Information.

## Data Availability

The data sets used and analyzed during the current study are available from the corresponding author at a reasonable request.

## Abbreviations

BMI: Body Mass Index
CI: Confidence Interval
DM: Diabetes
DPP4i: Dipeptidyl peptidase 4 inhibitors
Govt.: Government
HbA1c: Glycosylated hemoglobin
HI: Human insulin
I: Insulin
MI: Modern insulin/insulin analogue
SD: Standard deviation
SGLT-2: Sodium-Glucose-Linked Transporter-2
SU’s: Sulphonylureas

## Code availability

The collected data were analyzed using statistical tools in SPSS version 22.0 data analyzing software.
