# Supplementary material for: An observational multi-center study on type 2 diabetes treatment prescribing pattern and patient adherence to treatment
Source: Sci Rep. 2023 Dec 27;13:23037. doi: 10.1038/s41598-023-50517-2 (PMC10754831; doi:10.1038/s41598-023-50517-2)
Supplement: Supplementary file 1 — Supplementary Information. [file 41598_2023_50517_MOESM1_ESM.pdf]

PAKISTAN ENDOCRINE SOCIETY (PES) 2020 GUIDELINES FOR MANAGEMENT OF TYPE 2 DIABETES MELLITUS AND CARDIOMETABOLIC SYNDROME

Pakistan ranks 4th in the world with an estimated prevalence of 19.4 million people with diabetes as per IDF data published in 2019.[1] Pakistan faces health challenges in diabetes due to its high prevalence and its related complications.[2]

Pakistan Endocrine Society (PES) guidelines for type 2 diabetes mellitus (T2DM) are based on available local, regional and international scientific evidence including special considerations to affordability and availability of medicines in Pakistan and consensus statements by Guidelines committee of PES. (Recommendation) (Table-1).

These guidelines not only concentrate on diagnosis and management of T2DM but also provide a key to maintain referral system from primary to secondary and tertiary care and vice versa. Special emphasis has been given to develop the concept of multi-disciplinary team for the management of diabetes and hence chapters on nutrition, physical exercise and diabetes education have been included. The current document not only advocates glycemic control to reduce microvascular and macrovascular complications, but also highlights obesity as underlying risk factor for the development of T2DM. In addition, the document emphasizes recommendations for blood pressure (BP) and lipid control, the two most important risk factors for atherosclerotic cardiovascular disease (ASCVD) in addition to comprehensively managing micro and macro vascular complications. These recommendations will be revised periodically. Any major changes in the intervening period will be included as addendum/corrigendum.

It is important to highlight that these are only guidelines and hence individualized approach according to specific scenario is still the key to the management.

SUMMARY OF RECOMMENDATIONS FOR MANAGEMENT OF TYPE 2 DIABETES MELLITUS

INTRODUCTION

Pakistan Endocrine Society (PES) guidelines for type 2 diabetes mellitus (T2DM) are based on available local, regional and international scientific evidence including special considerations to affordability and availability of medicines in Pakistan and consensus statements by Guidelines committee of PES.

PES has made following recommendations on different issues pertaining to T2DM for Pakistan

RECOMMENDATIONS

RECOMMENDATION - 1: Risk of Developing T2DM

1.1- Pakistan ranks 4th in the world with an estimated prevalence of 19.4 million people with diabetes as per IDF data published in 2019.[1]

1.2- Population based screening for diabetes may be done using a locally validated screening test such as the Risk Assessment of Pakistani Individuals for Diabetes (RAPID) scoring system.[2] (Risk score attached).

High risk individuals are advised for laboratory testing.

People with a positive screening test should proceed to a diagnostic test as described in section 2. If the result of that test is normal, they should be advised on healthy lifestyle changes and the diagnostic test should be repeated every year.

RECOMMENDATION-2: Screening and Diagnosis of T2DM

2.1- Diabetes can be diagnosed on the basis of any of the following criteria:

- Fasting plasma glucose: ≥ 126 mg/dl (≥ 7.0 mmol/L) - Fasting defined as no caloric intake for minimal 8 hours, or plasma glucose: ≥ 200 mg/dl (11.1 mmol/L) in the presence of symptoms.
- Plasma glucose 2 hours after 75 gm glucose load ≥ 200 mg/dl (Modified oral glucose tolerance test although not reproducible
- HBA1c value of ≥ 6.5 DCCT (Diabetes control and complication trial) aligned and NGSP (National Glycohemoglobin Standardization Program) certified[5]
- Unless unequivocal symptomatic hyperglycemia is present, the diagnosis should be confirmed by repeat testing on a different day.

2.2- Fasting plasma glucose between 100-125 mg/dl or glucose value 2 hours after 75g OGTT between 140-199 mg/dl (>7.8 to 11 mmol/L) should receive a diagnosis of impaired glucose tolerance (IGT).

Diagnostic Criteria.[3,4]

	Normal (mg/dl)	IFG/IGT (MG/DL)	Diabetic (mg/dl)
FBS	<100	100-125	≥126
RBS/75gm OGTT	<140	140-199	≥200
HBA1C	<5.7	5.7-6.4	≥6.5

2.3- The above-mentioned tests should be performed in laboratory.

2.4- Asymptomatic individuals with a single abnormal test should have the same test repeated to confirm the diagnosis. On the other hand, if a patient has discordant results in two tests, the test result that is above the diagnostic cut point should be repeated. Symptomatic individuals do not need repetition of the abnormal test.

RECOMMENDATION - 3: Glycemic Targets &Assessment of Glycemic targets

Recommendation No 3.1: Glycemic Targets:[6]

Sub Category	Fasting blood sugar FBS mg/dl	Random blood sugar RBS mg/dl	Bed time sugar mg/dl	HbA1c %
Recent/without complications	80-120	80-160	100-140	6.5-7%
With CCF*, CKD†, CLD‡, Autonomic Neuropathy	100-140	120-180	120-180	7.0-7.5

Recommendation - 3.2: Assessment of Glycemic targets:

Recommendation - 3.2.1: HbA1c:

- Perform the HbA1C test at least two times a year in patients who have stable glycemic control.
- Perform the HbA1C test quarterly in patients whose therapy has changed or who are not meeting glycemic goals.
- For most non-pregnant T2DM patients a reasonable HbA1C goal is < 7%.
- For selected individual patients (short duration of diabetes, type 2 diabetes treated with lifestyle or metformin only, long life expectancy, or no significant cardiovascular disease) HbA1C goal such as 6.5%, maybe advised if this can be achieved without significant hypoglycemia.
- Less stringent A1C goals such as, 8% may be appropriate for patients with a history of severe hypoglycemia, limited life expectancy, advanced microvascular or macrovascular complications, extensive comorbid conditions, or long-standing diabetes.

Recommended- 3.2.2: Self-monitoring of blood glucose (SMBG):

Frequency of SMBG will vary according to the treatment regimen and affordability of patients. Adherence to the prescribed frequency should be emphasized whenever possible.

- Daily SMBG is superior to less frequent monitoring.
- After achieving target blood glucose SMBG can be done less frequently.
- Monitoring BG before going to bed at night should be done to prevent nocturnal hypoglycemia.

1. ***Low Intensity SMBG:*** Two times/week (pre breakfast +bedtime). For most controlled non-affording T2DM and geriatric patients (age > 70 years) controlled with or without co-morbid conditions.
2. ***Moderate Intensity SMBG***: Two times daily (pre breakfast + one post meals can be suggested). For newly diagnosed or un-controlled non affording T2DM and controlled affording T2DM plus geriatric patients.
3. ***High Intensity SMBG:*** SMBG can be done 4 to 5 times every day or alternate day until target blood glucose is achieved. Once target is achieved, SMBG can be done 3 times (pre breakfast and Pre- and Post-meal of major meal or other meals as required every 4th or 5th day or as required).
4. ***Intensive Intensity SMBG***: For pregnant women on MNT or on metformin a total of 14 readings per week including pre-breakfast and 1h PPG or 2h PPG are suggested.

RECOMMENDATION 4: Non pharmacological Management of Diabetes

Recommendation 4.1: Lifestyle modifications (LSM):[7]

The key components of lifestyle therapy include:

- Diabetes self-management education (DSME)
- Medical nutrition therapy (MNT) comprising of healthy eating patterns,
- Regular and adequate physical activity,
- Sufficient amount of sleep,
- Smoking cessation with avoidance of all tobacco products.

Recommendation 4.1.1: Diabetes self-management education:

- Persons with T2D must improve their lifestyle from the time of diagnosis to reach the metabolic targets as soon as possible. This can be achieved best assisted with an effective education program
- Patients with T2D should be referred to a diabetes education program at the time of diagnosis and the program should be conducted by a trained diabetes educator (where available).
- Set individualized target glycated hemoglobin (HbA1c) levels with the patient, and provide a level of care to achieve and maintain that target.
- Offer self-monitoring of blood glucose as an integral part of self-management, and agree when it should be performed and how it should be interpreted and acted upon. A glucometer with low CV (coefficient of variance) can be a reliable tool to be used.
- When starting insulin therapy, employ a structured training program with active dose titration.

Recommendation 4.1.2: Medical Nutrition Therapy (MNT):[8-11]

MNT should be started soon after diagnosis of T2DM by someone with training in nutrition therapy preferably by a registered dietitian (where available) and reviewed as per need.

- MNT should be aimed at achieving normoglycemia, providing adequate calorie intake.
- Simple sugars should be avoided. Food containing complex carbohydrate intake is recommended.

- High dietary fiber and whole grain containing foods should be encouraged.
- Non-calorie sweeteners (aspartame) may be used safely in moderate amounts.
- Lean protein, oily fish and vegetable consumption should be increased.
- Provide personalized diet plan in the form of printed diet charts while plate models can be used where necessary.

Recommendation 4.1.3: Regular and adequate physical activity:[12-14]

- Physical Activity (PA), is an effective intervention in improving glycemic control, blood pressure and lipid levels in addition to improving sense of well-being.
- If patient has not been active at all, start slowly and increase activity over a period of time. Simple walk for at least 30 min, 5 days a week is enough in initial phases or simple aerobics that increase heart rate to 60-70% of maximum (Maximum Heart Rate = 220 - age in years).
- Minimal but significant changes in lifestyle like using stairs instead of taking elevators, parking car at a distance from workplace, keep walking while having conversation on phone etc. can bring significant change in activity status of a person.
- Plenty of water should be taken to avoid dehydration. In extremes of weather, indoor alternative exercises are favored.

Recommendation 4.1.4: Adequate Sleep:[14,15]

- All patients should be advised to sleep on average approximately 6-7 hours every night.
- Six to 9 hours of sleep every night is associated with a reduction in cardiometabolic risk factors, whereas sleep deprivation aggravates insulin resistance, hypertension, hyperglycemia, and dyslipidemia and increases inflammatory cytokines.

Recommendation 4.1.5: Smoking Cessation:

- Smoking cessation is another important component of lifestyle therapy and involves avoidance of all tobacco products including pan, gatka, huqqa, niswar, shisha and e-cigarettes.
- It should be emphasized that smoking is associated with an increase in cardiometabolic risk factors including insulin resistance, hypertension, hyperglycemia, and dyslipidemia.
- Counselling to quit smoking should be done at each visit

Recommendation 4.1.6: Weight Reduction:[16,17]

- For weight reduction emphasis should be placed on lowering caloric intake and inducing weight loss for patients with type 2 diabetes who are overweight..
- A sustained weight loss of even 5 to 10 percent of initial body weight in overweight individuals can have a lasting beneficial impact on serum glucose, dyslipidemia, and hypertension.
- Physical activity, diet, and behavioral modification are important components to accomplish weight loss. Additional options for weight loss are medications and bariatric surgery.

RECOMMENDATION 5: Pharmacological Management of Diabetes[18-20]

- Pharmacological therapy should be considered if one fails to achieve glycemic targets with nonpharmacological therapy (MNT & Physical activity) within target days. This should not be more than one month provided blood glucose is monitored and not significantly elevated.
- Pharmacological treatment should be started right away if significant hyperglycemia is documented at time of

diagnosis.

- The choice of diabetes therapies must be individualized based on attributes specific to both patients and the medications themselves in addition to the patient's cardiac, cerebrovascular, and renal status.
- The choice of therapy also considers ease of use and affordability. The therapeutic regimen should be as simple as possible to optimize adherence.
- Any of the selected regimes should be evaluated every three months with HbA1c and SMBG.
- Visit could be scheduled at shorter interval if there is Glycemic variability or hyper/hypoglycemia anticipated in initial management.
- If HbA1c is not available, SMBG and/or lab records can be helpful.
- People with diabetes should be assessed for possible side effects of drugs including hypoglycemic events, weight gain, fluid retentions, hepatic or renal impairment or cardiovascular risks.
- They should also be assessed for co morbidities, drug adherence and psychosocial issues.

Recommendation 5.1: Initial monotherapy:

- Metformin should be prescribed to all patients along with lifestyle modifications, irrespective of their baseline BMI, if there are no contraindications. (E high, R strong).
- If metformin is contraindicated or is not tolerated, GLP1 agonists, SGLT2 inhibitors, DPP4 Inhibitors, sulphonylureas or insulin can be used as alternative.

Recommendation 5.2: Initial Combination Therapy:

- In newly diagnosed people with diabetes presenting with signs and symptoms of hyperglycemia or having HbA1c >8.5%, a second oral agent or insulin should be considered along with metformin. Initial combination of sub maximal doses of antihyperglycemic agents produces better and quicker response than maximum doses of monotherapy.

Recommendation 5.3: Initial Insulin Therapy:

Consider initiating insulin therapy (with or without additional agents) in patients with newly diagnosed type 2 diabetes who are symptomatic and/ or have A1C ≥ 10% (86 mmol/L) and/or blood glucose levels ≥ 300 mg/dL and if there is evidence of ongoing catabolism (weight loss).[18-20]

Insulin as initial therapy is recommended for treatment of T2DM in people who are:

- Unable to tolerate oral hypoglycemics or non-insulin injectables.
- In situation when there is suspicion of patient having Type 1 vs. 2 and confirmation is not possible.
- Being treated for acute complications of diabetes (DKA, HHS).
- Undergoing surgery.
- Unable to use oral hypoglycemics and non-insulin injectables due to allergies, renal or hepatic disorders in newly diagnosed patients with signs and symptoms of ketosis.
- Insulin can be categorized according to either duration of action ranging from rapid acting insulin to short acting, intermediate acting, long acting and very long acting insulin or their source as human or analogue insulin. The human insulin is less expensive than analogues, hence more affordable.
- The dose should be adjusted at regular intervals. Less expensive human insulin is beneficial in most of the cases particularly if comprehensive education about preventing, identifying and timely correction of hypoglycemia has been imparted.

- Required initial dose is 0.2 to 0.5 U/kg/day or 10 U/day. Obese people may need higher dose. Treatment should be graded to reach the targets and avoid unnecessary risk to patient (Hypoglycemia). General rule is start low and go slow if possible, in titration of Insulin.

Recommended approach to start insulin:

Step 1: In case of high fasting blood glucose (FBG), an intermediate acting human insulin or basal analogue insulin with a dose of 0.1 to 0.2U/kg or 10 U/day can be added at bedtime with the current oral therapy. The insulin dose may be titrated once or twice/week to reach the desired FBG. Analogue basal (ultralong acting) insulin can be given at fixed time of the day to have proper effect on fasting blood glucose.

Step 2: High post meal blood glucose should be controlled by bolus insulin, either by regular human insulin or by ultrashort acting insulin analogue with meal(s) and titrated every 48 to 72 hours to achieve the desired post-meal targets.

- Initial use of pre-mixed insulin regimens should ideally be avoided as it may not achieve the desired targets and put the patient at risk of fluctuating glycemic control. However, premixed insulin can be considered on individual basis where patients are unwilling to or unable to take basal bolus regimen. Insulin regimens like free mixing can also be considered for better management. These regimens require a vigilant follow up and patient's understanding of insulin use.
- Add GLP-1 receptor agonist to the basal insulin. The combination of GLP-1 receptor agonist and basal insulin is more effective in lowering glucose levels and has a lesser chance of weight gain and hypoglycemia as compared to the intensified insulin regimen. Also provides additional cardiovascular benefits to patient. However, cost is a major challenge in poor socioeconomic population.

Step 3: Intensive Insulin Therapy:

- If the HbA1c target is still not being met on basal insulin along with single injection of rapid-acting insulin before the largest meal of the day, proceed to a basal-bolus regimen with either 2 or 3 injections of rapid-acting insulin before each meal i.e. before breakfast, lunch and dinner. Insulin regimens like split mix and modified split mix can also be considered for better management. These regimens require a vigilant follow up and patient awareness about risk of hyper/hypoglycemia. These regimens require very intensive patient education..

RECOMMENDATION 6: Hypertension and Diabetes

High blood pressure is recognized as a major risk factor for CVD and CKD.

Recommendation 6.1.1: Monitoring of Blood pressure:

- Blood pressure should be measured at every clinic visit. Patients newly diagnosed with systolic blood pressure of ≥ 140 mmHg or a diastolic blood pressure of ≥ 90 mmHg should have blood pressure confirmed on a subsequent day.
- Blood pressure measurement should be measured by trained personnel. Standard protocol for blood pressure measurement must be followed i.e., in the seated position, with feet on the floor and arm supported at heart level, after 5min of rest. Cuff size should be appropriate for the upper arm circumference.

Recommendation 6.1.2: Blood Pressure (Targets):

- Systolic blood pressure (SBP) target should be <140 mmHg and diastolic blood pressure should be <90 mmHg in all people with diabetes and hypertension. There is limited evidence for the benefits of further lowering systolic blood pressure or diastolic blood pressure targets.
- If complications are present (additional risk factors and small vessel disease, particularly albuminuria), a tighter target may be appropriate.

Recommendation 6.1.3: Therapeutic Management Strategies:

- Patients with confirmed blood pressure readings of >140/90mmHg should be promptly initiated pharmacological

therapy, in addition to dietary changes (e.g. DASH Diet/ Sodium Intake) and life style modifications. Therapy must be titrated to achieve the desired goals.

- Lifestyle modifications consists of reducing excess body weight, increasing consumption of fruits and vegetables (4-5 servings per day), consuming low-fat dairy products (2-3 servings per day) and increasing activity levels.
- Sodium intake is restricted, the restriction should be the same for people with and without diabetes. The simplest strategy is not to add table salt to meals.
- For patients with diabetes and hypertension, ACE inhibitor/ARBs should be considered as initial therapy.
- If target blood pressure level is not achieved after 2-3 months, addition of either a calcium channel blocker, β-blocker or thiazide diuretic may be considered.
- Initial combination therapy may be needed when SBP is >20 mmHg and/or DBP is >10 mmHg above target, but this may vary with ethnicity and age.
- If ACE inhibitors, ARBs, or diuretics are used, serum creatinine and serum potassium levels should be monitored after10 days and then at 6th week. Ideally monitor creatinine every six to twelve months if it does not exceed more than 30% from its baseline.
- It is common to have an acute rise in serum creatinine of up to 30% within 2-5 days of initiating an ACEI or ARB, especially if the patient has CKD/CHF. These can be safely continued in these patients if the creatinine subsequently stabilizes at the higher level.
- If blood pressure remains uncontrolled despite good compliance to optimal doses of at least three antihypertensive agents of different class, one of which should be a diuretic, an evaluation for secondary hypertension should be considered.
- Additionally, antihypertensive effects of other medication such as SGLT 2 inhibitor, GLP Agonist and statins should be taken into account specially when starting them during same visit.

RECOMMENDATION - 7: Dyslipidemia and Diabetes:

High blood lipid levels are considered as a major cardiovascular risk factor and particularly high LDL cholesterol. All people with T2D and established CVD should start treatment with a statin (secondary prevention).

- **Recommendation 7.1:** Perform lipid profile including total cholesterol (TC), Low density Lipoprotein cholesterol (LDL-C), High density Lipoprotein Cholesterol (HDL-C) and Triglycerides (TG) at the time of diagnosis..
- **Recommendation 7.2:** Lifestyle modifications specially targeting weight reduction in overweight or obese people and modification of diet is the important part of management of dyslipidemia.
- **Recommendation 7.3:** All patients with type 2 diabetes over the age of 40 years or below if they have an additional cardiovascular risk factor should receive statin therapy and up-titrated to reach an LDL cholesterol target <70 mg/dL (1.8 mmol/L), despite any level of baseline LDL, unless a very clear risk is identified to withhold the therapy.
- **Recommendation 7.4:** All people with T2D and without established CVD who are ≥ 40 years old and have LDL cholesterol >100 mg/dL (2.6 mmol/L), should start treatment with a statin (primary prevention).
- **Recommendation 7.5:** If statin is not tolerated or a particular LDL-C goal is not achieved on statin alone, addition of a non-statin lipid-lowering agent can be considered.
- **Recommendation 7.6:** Target LDL cholesterol to be lowered by 50 percent of the baseline or less than 100mg/dl. In patients with atherosclerotic cardiovascular disease risk factors LDL cholesterol target <70 mg/dl is advisable.
- **Recommendation 7.7:** If triglycerides are high, more than 150mg/dl but less than 500mg/dl, strict lifestyle modifications, glycemic control and statins are recommended.

- **Recommendation 7.8:** If triglycerides are >500 to 1000 mg/dL (5.7-11.4 mmol/L) despite lifestyle changes and improved glycemic control should start a fibrate to prevent acute pancreatitis.
- **Recommendation 7.9:** When a health care provider considers that the patient needs statin therapy, it should be maintained lifelong.

RECOMMENDATION - 8: Antiplatelet Treatment

- **Recommendation 8.1:** Low dose aspirin therapy is an option in people with diabetes with increased CVD risk (Primary prevention). This includes family history of CVD, hypertension, smoking, dyslipidemia, or albuminuria and age more than 50 years.
- **Recommendation 8.2:** Aspirin should be given to all patients with established CVD disease. (Secondary prevention).
- **Recommendation 8.3:** It may not be recommended in people younger than 50 years without additional CVD risk factors.
- **Recommendation 8.4:** People intolerant to aspirin or if there is any contraindication, clopidogrel is an alternate option.
- **Recommendation 8.5:** Aspirin can be prescribed at a dose of 75-162 mg/day for both primary (high risk) and secondary prevention where indicated.
- **Recommendation 8.6:** Dual antiplatelet therapy (aspirin plus clopidogrel) is not indicated for primary prevention in T2DM.

RECOMMENDATION 9: Screening for Microvascular Complications::

Recommendations 9.1: Retinopathy screening

- All patients type 2 diabetes should have dilated eye examination by an ophthalmologist at diagnosis or at first visit to the clinic.
- If the screening for retinopathy is positive or if the patient has unexplained reduced visual acuity with or without retinopathy, the individual should be referred to an ophthalmologist.
- If no sign of retinopathy is present repeat examination annually. If retinopathy is present, frequency of examination should be suggested by ophthalmologist.
- Aspirin can safely be prescribed in patients with retinopathy as it does not increase the chances of retinal hemorrhage unless there is some other contraindication.

Recommendations 9.2: Nephropathy screening

- All patients with type 2 diabetes should be screened for microalbuminuria annually.
- Measure serum creatinine every six months to calculate eGFR once albuminuria is detected and/or when other risk factors are present (e.g., hypertension).
- Uncontrolled diabetes or hypertension, fever, infection, recent exercise or congestive cardiac failure may result in proteinuria without kidney disease.
- Two readings three months apart should be taken before making a diagnosis of nephropathy.
- Diabetic kidney disease (DKD, diabetic nephropathy) is identified when eGFR is <60 mL/min/1.73 m2 and/or albuminuria ≥30 mg/g creatinine
- Persistent albuminuria requires treatment with an ACE inhibitor or an ARB even in normotensive people after taking baseline serum creatinine and potassium.

- For patients with type 2 diabetes and chronic kidney disease, consider use of a sodium-glucose cotransporter 2 inhibitor or glucagon-like peptide 1 receptor agonist which has shown to reduce risk of chronic kidney disease progression, cardiovascular events, or both.
- In non-pregnant patients with diabetes and hypertension the first line anti hypertensives are ACEI or ARBs. Combination of these drugs with each other should be avoided due to increased incidence of hyperkalemia
- In selected high-risk patient, Serum creatinine and potassium should be rechecked after 10 days and 6 weeks in cases of newly prescribed ACEI/ARBs. It is common to have an acute rise in serum creatinine of up to 30% within 2-5 days of initiating an ACEI/ARB. These can be safely continued in patients if the creatinine subsequently stabilizes at the higher level.
- Good metabolic control is essential to delay the progression of nephropathy.
- Dietary proteins should be restricted to 0.8mg/kg / day if macroalbuminuria is present
- People should be referred to a nephrologist when they have DKD stage 4 or 5 (eGFR<30 mL/min/1.73 m2) or unexplained heavy proteinuria with or without hematuria in the absence of retinopathy or with short disease duration (e.g., other causes of renal disease) or with a rapid fall in the eGFR.

Recommendation 9.3: Neuropathy screening

- All people with diabetes require thorough assessment for peripheral neuropathy on presentation. Frequency of follow up assessment depends on presence of neuropathy and/or loss of protective sensations.
- Most common presenting complaints are pain, burning and tingling sensations. Almost 50% of patients may be asymptomatic. Identifying these insensate feet is important for prevention of foot ulcers.
- This assessment includes testing with 10 grams monofilament and any of the additional tests for pin prick, vibration or temperature sense to identify if the foot is at risk
- Medication with proven efficiency include duloxetine, gabapentin or pregabalin can be given as initial treatment. Additionally amitriptyline and nortriptyline can be offered with caution due to side effect profile.
- Other Secondary causes of Neuropathy must be evaluated in case of new onset neuropathy. In patients presenting with atypical or painful neuropathy, other causes should be excluded like, vit B12 deficiency, Renal disease, Vasculitis, thyroid disease, vitamin D Deficiency, neurotoxic medications, chronic inflammatory demyelinating neuropathy etc., by obtaining relevant tests.
- Take history regarding symptoms of autonomic neuropathy involving cardiovascular system, gastrointestinal tract including gastroparesis, genitourinary system including erectile dysfunction (ED) and offer appropriate treatment.

RECOMMENDATIONS 10: Screening for Macrovascular Disease

Recommendations 10.1: Screening for Coronary Artery Disease:

- Screen for coronary artery disease (CAD) when the patient has typical or atypical symptoms. (Chest pain, shortness of breath, orthopnea, paroxysmal nocturnal dyspnea etc.)
- Assess cardiovascular risk factors in all T2DM patients annually (Hypertension, dyslipidemia, smoking, obesity, family history of premature CVD etc.). Special attention should be paid to patient presenting with microvascular complication such as retinopathy / microalbuminuria as they might have silent macrovascular complications well.
- Offer aspirin and statin to patients who are at increased risk of CVD.
- Offer ACE inhibitors or ARBS to hypertensive diabetic patients with nephropathy.
- TZDs should be avoided in symptomatic patients with CHF

Recommendations 10.2: Screening for Peripheral Artery Disease (PAD):

- Screen for peripheral artery disease (PAD) by palpating the foot pulses and/ or measuring the SBP to calculate the ankle/brachial index.
- If symptoms of peripheral arterial disease are present refer the patient to secondary/tertiary care.
- All diabetic patients with non- healing ulcer having ABI <0.9 should be referred to secondary or tertiary centers for further evaluation of PAD by color duplex ultrasound followed by CT angiography, MR angiography or standard X-ray angiography, if required.
- All patients with diabetes and an ischemic foot ulcer should receive aggressive cardiovascular risk management including support for cessation of smoking, treatment of hypertension, control of hyperglycemia and prescription of a statin as well as low-dose aspirin or clopidogrel.
- Consider reevaluating the Diabetes regimen in these individual. SGLT 2 Inhibitors use should be assessed as they have potential of complication like amputation when used in patient with PVD.

RECOMMENDATION 11: Diabetes and Foot Care:

- Examine feet at each clinic visit to identify the presence of peripheral neuropathy, peripheral artery disease, previous healed ulcers, foot deformity, pre ulcerative signs, improper hygiene or foot wear.
- History of claudication or rest pain in lower limb should be taken. Inspect for color, temperature or edema. Palpation of peripheral pulses at each examination should be done.
- A risk category should be assigned (Table-1) for further preventive measures. Examination is also essential even in

the absence of symptoms.

- Assessment of neuropathy can be done with 10 gm. Monofilament for pressure perception, 128 Hz tuning fork for vibration sense and tactile sensation by cotton wool. Achilles tendon reflex should be examined.
- People should be referred to a vascular surgeon if they have severe intermittent claudication.
- Persons with diabetic foot ulcers should be referred to a diabetic foot clinic, where the treatment by a multidisciplinary specialized team will reduce the risk of amputation and the time to functional recovery

References

1. International Diabetes Federation. IDF Diabetes Atlas, 9th ed. Brussels, Belgium: 2019.
2. Riaz M, Basit A, Hydrie MZ, Shaheen F, Hussain A, Hakeem R, et al. Risk assessment of Pakistani individuals for diabetes (RAPID). Prim Care Diabetes 2012; 6:297-302. doi: 10.1016/j.pcd.2012.04.002.
3. Garber AJ, Handelsman Y, Grunberger G, Einhorn D, Abrahamson MJ, Barzilay JI, et al. Consensus statement by the American Association of Clinical Endocrinologists and American College of Endocrinology on the comprehensive type 2 diabetes management algorithm-2020 executive summary. Endocr Pract 2020; 26:107-39. Doi: 10.4158/CS-2019-0472.
4. American Diabetes Association. 2. Classification and Diagnosis of Diabetes: Standards of Medical Care in Diabetes-2019. Diabetes Care 2019; 42(Suppl 1):s13-28. doi: 10.2337/dc19-S002.
5. Aamir AH, Ul-Haq Z, Mahar SA, Qureshi FM, Ahmad I, Jawa A, et al. Diabetes Prevalence Survey of Pakistan (DPS-PAK): prevalence of type 2 diabetes mellitus and prediabetes using HbA1c: a population-based survey from Pakistan. BMJ Open 2019; 9:e025300. doi: 10.1136/bmjopen-2018-025300.
6. Basit A, Khan A, Khan RA. BRIGHT Guidelines on Self-Monitoring of Blood Glucose. Pak J Med Sci 2014; 30:1150-5. doi: 10.12669/pjms.305.6006.
7. Hoskin MA, Bray GA, Hattaway K, Khare-Ranade PA, Pomeroy J, Semler LN, et al. Prevention of Diabetes Through the Lifestyle Intervention: Lessons Learned from the Diabetes Prevention Program and Outcomes Study and its Translation to Practice. Curr Nutr Rep 2014; 3:364-78. doi: 10.1007/s13668-014-0094-2.
8. Evert AB, Boucher JL, Cypress M, Dunbar SA, Franz MJ, Mayer-Davis EJ, et al. Nutrition therapy recommendations for the management of adults with diabetes. Diabetes Care 2013; 36:3821-42. doi: 10.2337/dc13-2042.
9. Bergenstal RM, Johnson M, Powers MA, Wynne A, Vlajnic A, Hollander P, et al. Adjust to target in type 2 diabetes: comparison of a simple algorithm with carbohydrate counting for adjustment of mealtime insulin glulisine. Diabetes Care 2008; 31:1305-10. doi: 10.2337/dc07-2137.
10. Keogh JB, Clifton PM. Meal replacements for weight loss in type 2 diabetes in a community setting. J Nutr Metab 2012; 2012:918571. doi: 10.1155/2012/918571.
11. Gonzalez-Campoy JM, St Jeor ST, Castorino K, Ebrahim A, Hurley D, Jovanovic L, et al. Clinical practice guidelines for healthy eating for the

prevention and treatment of metabolic and endocrine diseases in adults: cosponsored by the American Association of Clinical Endocrinologists/the American College of Endocrinology and the Obesity Society. Endocr Pract 2013; 19(Suppl 3):1-82. doi: 10.4158/EP13155.GL.
12. Balducci S, Alessi E, Cardelli P, Cavallo S, Fallucca F, Pugliese G. Effects of different modes of exercise training on glucose control and risk factors for complications in type 2 diabetic patients: a meta-analysis: response to Snowling and Hopkins. Diabetes Care 2007; 30:e25. doi: 10.2337/dc06-2495.
13. Manders RJ, Van Dijk JW, van Loon LJ. Low-intensity exercise reduces the prevalence of hyperglycemia in type 2 diabetes. Med Sci Sports Exerc 2010; 42:219-25. doi:10.1249/MSS.0b013e3181b3b16d.
14. Church TS, Blair SN, Cocreham S, Johannsen N, Johnson W, Kramer K, et al. Effects of aerobic and resistance training on hemoglobin A1c levels in patients with type 2 diabetes: a randomized controlled trial. JAMA 2010; 304:2253-62. doi: 10.1001/jama.2010.1710.
15. McNeil J, Doucet É, Chaput JP. Inadequate sleep as a contributor to obesity and type 2 diabetes. Can J Diabetes 2013; 37:103-8. doi: 10.1016/j.jcjd.2013.02.060.
16. Cappuccio FP, Cooper D, D'Elia L, Strazzullo P, Miller MA. Sleep duration predicts cardiovascular outcomes: a systematic review and meta-analysis of prospective studies. Eur Heart J 2011; 32:1484-92. doi: 10.1093/eurheartj/ehr007.
17. Mechanick JI, Garber AJ, Handelsman Y, Garvey WT. American Association of Clinical Endocrinologists' position statement on obesity and obesity medicine. Endocr Pract 2012; 18:642-8. doi: 10.4158/EP12160.PS.
18. Garvey WT, Mechanick JI, Brett EM, Garber AJ, Hurley DL, Jastreboff AM, et al. American Association of Clinical Endocrinologists and American College of Endocrinology comprehensive clinical practice guidelines for medical care of patients with obesity. Endocr Pract 2016; 22(Suppl 3):1-203. doi: 10.4158/EP161365.GL.
19. American Diabetes Association. Index. Diabetes Care 2020; 43(Suppl 1):s1-212. Doi: 10.2337/dc20-in01
20. Garber AJ, Handelsman Y, Grunberger G, Einhorn D, Abrahamson MJ, Barzilay JI, et al. Consensus Statement by The American Association of Clinical Endocrinologists and American College of Endocrinology on The Comprehensive Type 2 Diabetes Management Algorithm - 2020 Executive Summary. Endocr Pract 2020; 26:107-39. doi: 10.4158/CS-2019-0472.

CHAPTER-4
PHARMACOLOGICAL MANAGEMENT OF DIABETES

Overview:

- The choice of pharmacological agent must be individualized, based on attributes specific to both patients and the medications.
- The choice of therapy also depends on the patient's age, BMI, duration of disease, glycemic status, risk of hypoglycemia, cardiac, cerebrovascular, renal function and financial status
- The choice of therapy should take into consideration ease of use and availability. The therapeutic regimen should be as simple as possible to optimize adherence.
- Comorbidities must be managed for comprehensive care, including management of lipid and blood pressure abnormalities and treatment of other related conditions.
- Any of the selected regimes should be evaluated every three months with HbA1c and SMBG. If HbA1c is not available, SMBG and/or lab records can be helpful. Patients should be assessed for possible side effects of drugs, including hypoglycemic events, weight gain, fluid retention, hepatic or renal impairment or cardiovascular risks. They should also be assessed for co morbidities, drug adherence and psychosocial issues.[1]
- Metformin should be prescribed to all patients along with lifestyle modifications, irrespective of their baseline BMI, if there are no contraindications.[2] If metformin is contraindicated or is not tolerated, GLP1 agonists or SGLT2 inhibitors can be prescribed as preferred agents. DPP4 Inhibitors, sulphonylureas or alpha glucosidase inhibitors can be used as alternatives.[3]
- In newly diagnosed patients with T2DM presenting with signs and symptoms of hyperglycemia and having an HbA1c >8.5%, a second oral agent or insulin should be considered along with metformin. Initial combination of sub maximal doses of antihyperglycemic agents produces better and quicker response than maximum doses of monotherapy.[3-5]

Factors Influencing Management Strategies

CLINICAL FACTORS

- Age
- Weight
- Degree of hyperglycemia
- Risk of hypoglycemia / hypoglycemia unawareness
- Presence of any co-morbidity/complication
- Socio-economic status
- Individual preference

PHARMACOLOGICAL FACTORS

- Efficacy in glycemic control
- Risk of hypoglycemia
- Risk of weight gain

- Drug interactions
- Side effects
- Cost and availability
- Cardiovascular benefits.
- Renal benefits

RATIONALE AND EVIDENCE

Available Therapeutic Agents in Pakistan

Metformin

- Metformin is currently the drug of first choice for the treatment of hyperglycemia in DM, without stimulating insulin secretion, promoting weight gain, or causing hypoglycemia.[2,3]
- Metformin is an insulin-sensitizer, which causes reduction in insulin resistance and a significant decrease in plasma fasting insulin levels. Metformin monotherapy can reduce HbA1c by 1.1%.[6]
- It also provides benefits of weight stability or slight weight reduction.[7]
- It is generally well tolerated. Most commonly reported side effects are anorexia, nausea, diarrhea and a metallic taste. These effects can be minimized if metformin is taken with meals.
- Lactic acidosis is the only serious side effect. However, its risk incidence is extremely low.[8]
- It is contraindicated in CKD Stage 4 and 5 (eGFR<30).9 If eGFR is not available metformin should be discontinued at serum creatinine>1.5mg/dl in men and > 1.4 mg/dl in women.
- Metformin is excreted by the kidneys. The reduction in renal clearance of metformin is considered as an important risk factor for lactic acidosis it should be started at a low dose and titrated upwards until the required glycemic targets are achieved or another oral agent is added in regime.
- It may also be associated with Vit B12 deficiency in some cases with long term use.
- The maximum dose is up to 2550 mg in divided doses. It should be started at a low dose, typically 500mg twice daily, with upward titration if desirable control of hyperglycemia is not achieved. The drug is well tolerated.

SGLT2 Inhibitors:

- Sodium-glucose co- transporters (SGLT2) are present in proximal tubules of kidneys. Kidneys filter glucose freely, 90% of which is reabsorbed in the proximal tubules by the action of SGLT2.
- SGLT2 inhibitors lower HBA1c by 0.7-1%.[10] Available SGLT2 agents in Pakistan are dapagliflozin (5 and 10 mg) and empagliflozin (10 and 25 mg).
- Dose of dapagliflozin is 10 mg daily, but it is recommended to start with 5mg initially. Dose of empagliflozin is 10 mg daily, but higher dose of 25 mg daily can be used.
- The main side effects are increased incidence of genital mycotic infections and urinary tract infections.
- Efficacy of SGLT2i is decreased in chronic kidney disease and is contraindicated in patients with eGFR less than <30.[2,3,10]

GLP 1 agonists

- Glucagon-like peptide 1 (GLP-1) is known for the 'incretin effect', which results in a glucose dependent increase in insulin secretion and suppression of glucagon secretion from the pancreas.

- GLP-1RAs improve glycemic control, reduce patient weight and improve patient-reported outcomes when administered as monotherapy or add-on therapy to other glucose-lowering drugs.
- GLP-1RAs reduce, or at least not increase, the risk of major cardiovascular events.
- GLP-1RAs are generally well tolerated with a very low intrinsic risk of hypoglycemia.
- Also suppress pancreatic glucagon output, retard gastric emptying and diminish appetite. This usually results in weight reduction.
- Are expensive and administered subcutaneously on a daily or weekly basis.
- Can reduce HbA1c by about 0.6% to 0.8% when used as monotherapy.[11,12]
- Once-weekly administration of GLP-1RAs may improve treatment adherence and satisfaction relative to more frequent treatment.
- Patients should be advised to seek medical care if they experience unexplained persistent severe abdominal pain.
- Available agents in Pakistan are Liraglutide (Prefilled, multidose pen that delivers doses of 0.6mg, 1.2mg, or 1.8 mg, to be taken once a day) and Dulaglutide (1.5mg single dose pen, taken once weekly).

Dipeptidyl Peptidase IV inhibitors (DPP4 Inhibitors)

- The oral dipeptidyl peptidase IV (DPP-4) inhibitors or incretin enhancers, increase circulating concentrations of active GLP-1 and GIP.[13]
- Lower HbA1c by approximately 0.5-1.0% and are weight neutral.[13,14]
- Proven efficacy when combined with metformin, sulfonylurea or both metformin and sulfonylurea.
- Low risk of hypoglycemia.
- Recent cardiovascular studies have shown that these agents do not increase the CV risk.[13,14]

Sulphonylureas (SU)

- SUs reduces plasma glucose levels by enhancing insulin secretion, with an average A1c reduction of 1.5%.[15]
- The major adverse side effect is hypoglycemia. The risk is higher in renal impairment, liver cirrhosis and in the elderly. Weight gain is also a common side effect.[13]
- Second generation SUs (gliclazide, glimepiride) have a lower risk of hypoglycemia and weight gain.
- Highly protein bound therefore administration of drugs like non-steroidal anti-inflammatory drugs (NSAIDs), antithyroid drugs, sulpha drugs, anticoagulants and α-blockers can displace them, increasing the risk of hypoglycemia.

Alpha glucosidase inhibitor (AGI)

- AGIs are Saccharides which act as competitive inhibitors of enzymes required to digest carbohydrates, including starch and table sugar, thus controlling postprandial hyperglycemia.
- Can reduce HbA1c by 0.2%.[16]
- Major side effects are bloating and flatulence. Hence usually not well tolerated. Side effects can be avoided by slow titration of dosage.

Thiazolidinedione (TZDs)

- These insulin sensitizers are PPAR gamma agonists.
- Major side effects include edema, weight gain, risk of congestive heart failure (CHF) and increased risk of fractures. This significantly limits their clinical use.[17]
- Have conflicting findings regarding myocardial infarction (MI) risk. However, they should not be used in NYHA class 2 patients.[2,3,17]
- Inconclusive evidence for their association with bladder cancer.[17]
- The starting dose of Pioglitazone is 15 mg /day and can be titrated to maximum dose of 45 mg/day.

Repaglinide

- Relatively short-acting stimulator of insulin secretion (<6 hours).
- Acts by binding to ATP dependent potassium channels on pancreatic beta cells.[18]
- The main risk is hypoglycemia and weight gain.
- Mainly excreted through hepatic route, hence is safe in renal compromised patients.[18]

Insulin

Insulin therapy is often required in people suffering from type 2 diabetes to optimize blood glucose control.[3] Insulin therapy in type 2 diabetes is recommended for patients who are:

- Unable to reach glycemic targets with lifestyle modification (diet and exercise) or with a maximum dose of oral hypoglycemics or noninsulin injectables, e.g. Glucagon like peptide-1 (GLP-1) receptor agonist
- Unable to tolerate oral hypoglycemics or non-insulin injectables.
- Being treated for acute complications of diabetes (DKA, HHS).
- Undergoing surgery.
- Unable to use oral hypoglycemics and non-insulin injectables due to allergies, renal or hepatic disorders.

Consider initiating insulin therapy (with or without additional agents) in patients with newly diagnosed type 2 diabetes, who are symptomatic and/ or have A1c ≥ 10% (86 mmol/L) and/or blood glucose levels ≥ 300 mg/dL and if there is evidence of ongoing catabolism (weight loss).[19,20]

Insulin can be categorized according to either duration of action ranging from rapid acting insulin to short acting, intermediate acting, long acting and very long acting insulin or their source as human or analogue insulin.

- The human insulin is less expensive than analogues, hence more affordable.
- The dose should be adjusted at regular intervals. Less expensive human insulins are beneficial in most of the cases particularly if comprehensive education about preventing, identifying and timely correction of hypoglycemia has been imparted.
- Different types of insulin and their duration of action are discussed in the table given below

How to initiate insulin in people with T2DM?

If Fasting Blood Glucose Levels are High:

- Oral hypoglycemic agents can be used to control blood glucose levels in combination with basal insulin.[2,3,19]
- A single dose of intermediate-acting insulin NPH or long-acting insulin Glargine U-100 or Detemir can be added at

bedtime with the current oral therapy.[18,19]

- To prevent hypoglycemia, it is advised to initiate insulin with a starting dose of 0.2 units/kg, while adjusting the dose by increasing 2 units every 3 days based on the fasting blood glucose levels until the desired target is achieved.[18,19]
- SMBG should be done at least twice daily, usually before breakfast and before bedtime, but more frequent SMBG is recommended to meet goals of the therapy.[19,20]

If only Post-Prandial Blood Glucose Levels are High:

The following options can be considered

- Continue the bedtime NPH injection and add a second injection of NPH before breakfast at a dose of 0.2 units per kg.[19,20] Metformin, SGLT2 inhibitors, DPP-4 may need to be continued. Addition of GLP-1 or SGLT2 inhibitors may help to improve control in patients with suboptimal glycemic levels requiring higher insulin doses, and may reduce the amount of insulin required in these patients.[20]
- Add rapid-acting or short-acting insulin before the largest meal of the day. Initiate with a starting dose of approximately 4 units,[18] while adjusting the dose by 2 units every 3 days until the desired target is achieved.[21]
- Add once daily dose of Degludec+Aspart (70/30) with largest meal of the day.
- Switch to pre-mixed or free-mix (R and NPH) insulin twice a day before breakfast and dinner.
- Add GLP-1 receptor agonist to the basal insulin. The combination of GLP-1 receptor agonist and basal insulin is more effective in lowering glucose levels and has a lesser chance of weight gain and hypoglycemia as compared to the intensified insulin regimen. However, cost is a major challenge in low socioeconomic countries like Pakistan.

Intensive Insulin Therapy:

If the HbA1c target is still not being met on basal insulin along with single injection of rapid-acting insulin before the largest meal of the day, proceed to a basal-bolus regimen with either 2 or 3 injections of rapid-acting insulin before each meal i.e. before breakfast, lunch and dinner.[21]

Example of Intensive insulin regimen by using rapid acting insulin or intermediate or long acting insulin in 70 kg man with type 1 diabetes. Assume he is consuming 75g carbohydrate at breakfast, 60g at lunch, and 90g at dinner.

Insulin	Pre breakfast	Pre lunch	Pre dinner	Bed time
**Rapid acting insulin analog lispro, aspart, glulisine**	6U	4U	6U	—
**NPH**	12U	0U	8U	
**OR**				
**Rapid acting insulin analog**	5U	4U	6U	—
**Insulin glargine OR degludec OR**	—	—	—	16U
**Insulin detemir**		—	—	16 U

- The dose of rapid acting analogs can be raised by 1 or 2 unit if extra carbohydrate (15-30g) is ingested or if premeal blood glucose is >170mg/dl.
- The rapid acting insulin can be mixed in the same syringe with NPH insulin.
- Insulin glargine or insulin detemir must be given as a separate injection.

Table: Insulin Commercially Available in Pakistan

Type of Insulin	**Brand Name**	**Manufacturer**	**Dosage form**
Ultra-short acting/ Rapid-acting Insulin analogs (Bolus)			
Lispro	**Humalog**	**Eli Lilly**	Vial/Cartridge/Pen
Aspart	**NovoRapid**	**Novo Nordisk**	Vial/Cartridge/Pen
Glulisine	**Apidra**	**Sanofi Aventis**	Vial/Pen
Short-acting/Regular Human Insulin (Bolus)			
Regular or Insulin R	**Humulin-R**	**Eli Lilly**	Vial
	**Actrapid**	**Novo Nordisk**	Vial/Cartridge
	**Insuget-R**	**Getz Pharma**	Vial
	**Innogen-R**	**Pharm Evo**	Vial
Intermediate-acting Insulin (Basal)			
NPH (Neutral Protamine Hagedorn)	**Humulin-N**	**Eli Lilly**	Vial
	**Insulatard**	**Novo Nordisk**	Vial/Cartridge
	**Insuget-N**	**Getz Pharma**	Vial
	**Innogen-N**	**Pharm Evo**	Vial
Long-acting Insulin Analogs (Basal)			
Insulin Glargine (U-100)	**Lantus**	**Sanofi Aventis**	Vial/Pre-filled pen
Insulin Detemir	**Levemir**	**Novo Nordisk**	Pre-filled pen
Ultra-Long acting Insulin Analogs (Basal)			
Insulin Glargine (U-300)	**Toujeo (1.5)**<br>**Toujeo Max (3 ml)**	**Sanofi Aventis**	Pre-filled pen
Pre-mixed Human Insulin (Intermediate-acting NPH + Short-acting Regular R) (70% N 30% R)			
NPH + Regular	**Humilin-70/30**	**Eli Lilly**	Vial/Cartridge
	**Mixtard-70/30**	**Novo Nordisk**	Vial/Cartridge
	**Insuget 70/30**	**Getz Pharma**	Vial
	**Innogen 70/30**	**Pharm Evo**	Vial
Pre-mixed Analogs [NPL (Neutral ProtamineLispro) + Lispro (Ultra-short acting Analog)]			

NPL + Lispro	**Humalog Mix 25**	**Eli Lilly**	KwikPen/Cartridge
	**Humalog Mix 50**	**Eli Lilly**	
Pre-mixed Analogs [70% Insulin Aspart Protamine + 30% Insulin Aspart (Ultra- short acting Analog)]			
Aspart Protamine + Aspart	**Novo Mix 30**	**Novo Nordisk**	FlexPen
	**Novo Mix 50**		
**Pre-mixed Ultra-long acting and Ultra-short acting Analogs (70% Insulin Degludec and 30% Insulin Aspart)**			
Degludec + Aspart	**Ryzodeg**	**Novo Nordisk**	Flex Pen
Insulin+GLP-1 combination			
Xultrophy	3.6mg Liraglutide/ml	100U degludec/ml	

Table : Insulin Pharmacodynamics					
**Type of Insulin**	**Onset**	**Peak**	**Effective Duration**	**Meal Relation**	**Color**
Ultra-short acting/ Rapid-acting Insulin analogs (Bolus)					
Lispro<br>Aspart<br>Glulisine	< 15 mins	1 Hr	2-4 Hrs	15 mins or just before/ after meal	Clear
Short-acting/Regular Human Insulin (Bolus)					
Regular or Insulin R	0.5-1 Hr	2-3 Hrs	3-6 Hrs	30-45 mins before meal	Clear
Intermediate-acting Insulin (Basal)					
NPH	2-4 Hrs	4-10 Hrs	10-16 Hrs	Not related with meal. Once, twice or thrice daily (6)	Cloudy
Long-acting Insulin Analogs (Basal)					
Insulin Detemir	0.8-2 Hrs (Dose Dependent)	Relatively Flat	24 Hrs	Not related to meal Once daily - Morning or Evening. Also can be given twice a day. (1)	Clear
Insulin Glargine (U-100)	2-4 Hr	Relatively Flat	20-24 Hrs		
Ultra-Long acting Insulin Analogs (Basal)					
Insulin Glargine (U-300)	2-4 Hr	Relatively Flat	>24 Hrs	Not related to meal. Once daily - Morning or Evening.	Clear
Pre-mixed Human Insulin (Intermediate-acting NPH + Short-acting Regular R) (70% N 30% R)					
NPH + Regular	0.5-1	Dual	10-16 Hrs	30-45 mins before meal	Cloudy
Pre-mixed Analogs [NPL (Neutral ProtamineLispro) + Lispro (Ultra-short acting Analog)]					
NPL + Lispro	< 15 Min	Dual	10-16 Hrs	15 min or just before/ after meal	Cloudy
Pre-mixed Analogs [70% Insulin Aspart Protamine + 30% Insulin Aspart (Ultra- short acting Analog)]					
Aspart Protamine + Aspart	<15 Min	Dual	15-18 Hrs	15 min or just before/ after meal. Can be given once, twice or thrice daily (7,8)	Cloudy
Pre-mixed Ultra-long acting and Ultra-short acting Analogs (70% Insulin Degludec and 30% Insulin Aspart)					
Degludec + Aspart	< 15 mins	Dual	>24 Hrs	15 min or just before/ after meal	Clear

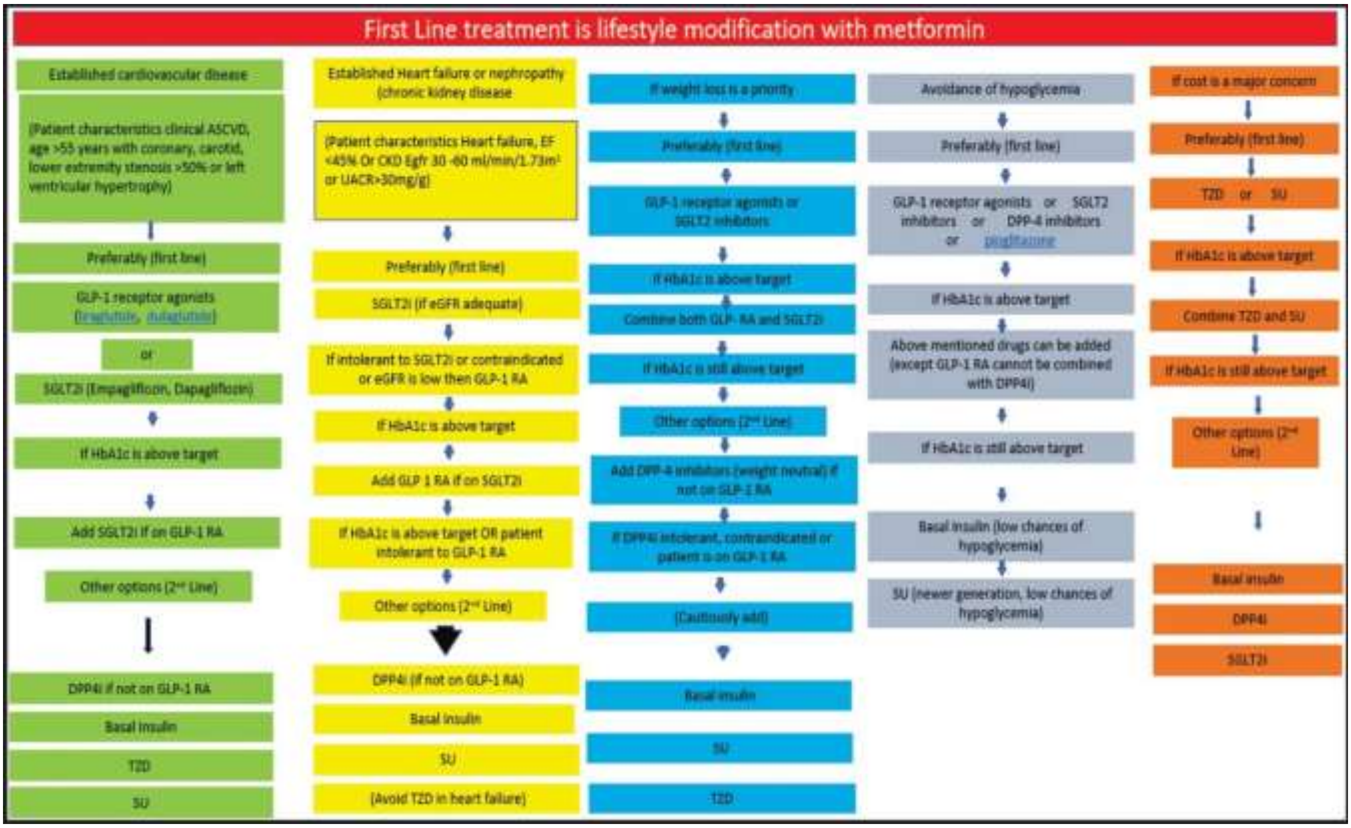

Insulin Initiation

Visit 1

Discuss need for insulin*, including *barriers to overcome
Review barriers. Adjust oral anti-diabetics Home BG testing for 2-4 wk., glucose log book

Visit 2

Review log book. Prescribe insulin. Demonstrate/teach use of insulin pen. Resources. Handouts on injection sites, dealing with low Blood Sugar, diet counselling

Visit 3

Review injection technique, review log, adjust insulin dose, Address patient concerns

Insulin Initiation

Insulin may be used at almost any stage of diabetes

<u>In primary care, consider insulin if:</u>

- Using 2 or more oral glucose lowering agents at or near maximal doses
- Diet, activity and medication have been reviewed and modified to minimize contributing factors
- Hyperglycemic Symptoms (wt. loss, polyuria, polydipsia)

AND

- A1C persistently > 8.5% (> 3-6 months) or
- A1C >10% and hyperglycemic symptoms (wt. loss, polyuria, polydipsia)

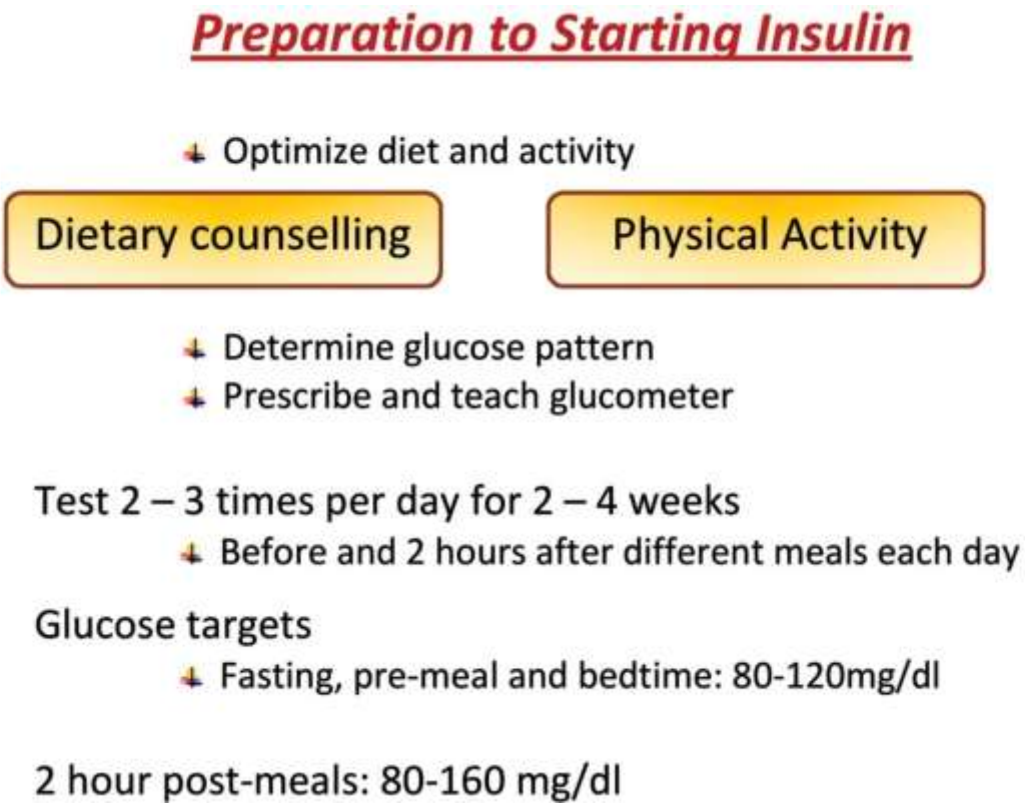

THREE DIFFERENT OPTIONS FOR INITIATION OF INSULIN

OPTION A

Basal added to Oral Agents	Start Insulin	Titration	Short Term Follow-up (1-4 week)	Long Term Follow-up (After 3 months)
Continue other oral agents initially (until glucose control improves with insulin)	**NPH (Humulin N, Insulatard]**<br>• least expensive, reasonable first choice for most T2DM<br><br>**Glargine (Lantus)**<br>• less nocturnal hypoglycemia, for patients who are prone to hypoglycemia<br><br>**Detemir (Levemir)**<br>• less nocturnal hypoglycemia, for patients who are prone to hypoglycemia, less weight gain	• Starting dose: 10-16 units OD at bedtime<br>• Test glucose 1-2 times per day: before breakfast and bedtime<br>• Increase basal insulin by 2 units every 3-5 days until fasting glucose is in target (FBG 80-120mg/dl)	• Fasting glucose still elevated<br>• Continue to increase dose FBG in target - No further increase<br>• A1C in 3 months	• Review A1C and glucose records after 3 months<br>• If A1C above target, consider intensifying insulin

OPTION B

Bolus Insulin with Meals	Titration	Testing & Insulin Adjustment	Short Term Follow-up (1-4 week)	Long Term Follow-up (After 3 months)
**Regular (Humulin R, Actrapid)**<br>• Lower cost<br>• Should be given 30-40 minutes before a meal<br>• Reasonable first choice for patients with consistent lifestyle, who do not require flexibility in their diet/ activity<br><br>**Rapid Acting (Aspart [Novorapid], Lispro [Humalog], Glulisine [Apidra])**<br>• Greater cost<br>• Must be given within 10-15 minutes before meal (may be given during or immediately after meal in some cases)<br>• Better choice for patients who desire flexibility in their diet and activity	• Start with 4-6 units before largest meal<br>• Increase by 1 unit every 2-3 days until 2-hr post-meal glucose is in target	• Test glucose 2 hours after meal(s)<br>• Increase bolus insulin by 1 – 2 units every 3 – 5 days until PPG <180 mg/dl	• 2-hour post-meal(s) glucose > 180 mg/dl – increase bolus insulin by 1 – 2 units every 3 – 5 days until PPG < 180 mg/dl	• If post-prandial glucose still elevated, consider increasing bolus doses or refer to endocrinology/ internal medicine

OPTION C

Pre-mixed Insulin	Titration	Testing & Insulin Adjustment	Short Term Follow-up (1-4 week)	Long Term Follow-up (After 3 months)
• Pre-mixed insulins<br>• Human Premix (30/70)<br>• Analogue Premix (Novomix-30, HumalogMix-25, Mix-50)	• Start with major meals at 0.3u/kg/day divided doses Split dose 50/50 or 70/30, depending on largest meal Titrate dose for glucose covered by NPH<br>• Increase 2 units every 2-3 days until target reached	• Test glucose 2 per day: Before Breakfast and Supper<br>• Increase pre-mixed insulin by 2 units every 3-5 days until pre-meal glucose in targetrange	• Pre-mixed Insulin BID<br>• Pre-breakfast glucose elevated – increase supper insulin<br>• Pre-supper glucose elevated – increase breakfast insulin& avoid afternoon snacking	• Review A1C and glucose records after 3 months If A1C above target, consider intensifying insulin<br>• Review diet and activity (more consistency, avoid simple carbs – especially at lunch)<br>• Consider switching to Basal/Bolus (depends on patient)

Few key points to go over with the patient before increasing the insulin dose:

Patient who are already on insulin and still have uncontrolled diabetes, before increasing insulin dose further, exercise and dietary compliance must be emphasized. Patient should be inquired about the timing of insulin injection, dosing of insulin and proper storage of insulin. Patient should also be asked about insulin injection technique. The areas where insulin should be injected are abdomen, anterior and lateral aspect of thighs, buttocks or sparingly the tricep fold of arms. Inspection of site of Insulin injection is of utmost importance in these patients for redness, swelling, lipohypertrophy (hypertrophy of subcutaneous tissue due to injecting insulin at the same site) and lipodystrophy (immune mediated disfiguring atrophy of tissues). Insulin in not effectively absorbed if any of these above-mentioned complications are present. Before changing insulin dose above issues need to be addressed if present. To avoid these complications best approach is to use sterile technique for insulin administration. Injection site should be rotated on frequent basis and body area for injection should also be rotated on as needed basis.

References

1. Garber AJ, Abrahamson MJ, Barzilay JI, Blonde L, Bloomgarden ZT, Bush MA, et al. Consensus Statement by the American Association of Clinical Endocrinologists and American College of Endocrinology on the comprehensive type 2 diabetes management algorithm--2016 executive summary. Endocr Pract 2016; 22:84-113. doi: 10.4158/EP151126.CS.
2. American Diabetes Association. 9. Pharmacologic Approaches to Glycemic Treatment: Standards of Medical Care in Diabetes-2020. Diabetes Care 2020; 43(Suppl 1):s98-110. doi: 10.2337/dc20-S009.
3. Garber AJ, Handelsman Y, Grunberger G, Einhorn D, Abrahamson MJ, Barzilay JI, et al. Consensus Statement by the American Association of Clinical Endocrinologists and American College of Endocrinology on the comprehensive type 2 diabetes management algorithm - 2020 executive summary. Endocr Pract 2020; 26:107-39. doi: 10.4158/CS-2019-0472.

4. Yki-Järvinen H. Thiazolidinediones. N Engl J Med 2004; 351:1106-18. doi: 10.1056/NEJMra041001.
5. Dormandy JA, Charbonnel B, Eckland DJ, Erdmann E, Massi-Benedetti M, Moules IK, et al. Secondary prevention of macrovascular events in patients with type 2 diabetes in the PROactive Study (PROspective pioglitAzone Clinical Trial In macroVascular Events): a randomised controlled trial. Lancet 2005; 366:1279-89. doi: 10.1016/S0140-6736(05)67528-9.
6. International Diabetes Federation. IDF Diabetes Atlas, 9th ed. Brussels, Belgium: 2019.
7. Lipska KJ, Bailey CJ, Inzucchi SE. Use of metformin in the setting of mild-to-moderate renal insufficiency. Diabetes Care 2011; 34:1431-7. doi: 10.2337/dc10-2361.
8. Salpeter SR, Greyber E, Pasternak GA, Salpeter EE. Risk of fatal and nonfatal lactic acidosis with metformin use in type 2 diabetes mellitus. Cochrane Database Syst Rev 2010; 2010:CD002967. doi: 10.1002/14651858.CD002967.pub4.
9. Beulens JW, Hart HE, Kuijs R, Kooijman-Buiting AM, Rutten GE. Influence of duration and dose of metformin on cobalamin deficiency in type 2 diabetes patients using metformin. Acta Diabetol 2015; 52:47-53. doi: 10.1007/s00592-014-0597-8.
10. Rabizadeh S, Nakhjavani M, Esteghamati A. Cardiovascular and Renal Benefits of SGLT2 Inhibitors: A Narrative Review. Int J Endocrinol Metab 2019; 17:e84353. doi: 10.5812/ijem.84353.
11. Castellana M, Cignarelli A, Brescia F, Perrini S, Natalicchio A, Laviola L, et al. Efficacy and safety of GLP-1 receptor agonists as add-on to SGLT2 inhibitors in type 2 diabetes mellitus: A meta-analysis. Sci Rep 2019; 9:19351. doi: 10.1038/s41598-019-55524-w.
12. Lyseng-Williamson KA. Glucagon-Like Peptide-1 Receptor Analogues in Type 2 Diabetes: Their Use and Differential Features. Clin Drug Investig 2019; 39:805-19. doi:10.1007/s40261-019-00826-0.
13. White WB, Pratley R, Fleck P, Munsaka M, Hisada M, Wilson C, et al. Cardiovascular safety of the dipetidyl peptidase-4 inhibitor alogliptin in type 2 diabetes mellitus. Diabetes Obes Metab 2013; 15:668-73. doi: 10.1111/dom.12093.
14. Deacon CF. Physiology and Pharmacology of DPP-4 in Glucose Homeostasis and the Treatment of Type 2 Diabetes. Front Endocrinol (Lausanne) 2019; 10:e80. doi:10.3389/fendo.2019.00080.
15. Forst T, Hanefeld M, Jacob S, Moeser G, Schwenk G, Pfützner A, et al. Association of sulphonylurea treatment with all-cause and cardiovascular mortality: a systematic review and meta-analysis of observational studies. Diab Vasc Dis Res 2013; 10:302-14. doi: 10.1177/1479164112465442.
16. Chiasson JL, Josse RG, Gomis R, Hanefeld M, Karasik A, Laakso M. Acarbose for prevention of type 2 diabetes mellitus: the STOP-NIDDM randomised trial. Lancet 2002; 359:2072-7. doi: 10.1016/S0140-6736(02)08905-5.
17. Levin D, Bell S, Sund R, Hartikainen SA, Tuomilehto J, Pukkala E, et al. Pioglitazone and bladder cancer risk: a multipopulation pooled, cumulative exposure analysis. Diabetologia 2015; 58:493-504. doi: 10.1007/s00125-014-3456-9.
18. Guardado-Mendoza R, Prioletta A, Jiménez-Ceja LM, Sosale A, Folli F. The role of nateglinide and repaglinide, derivatives of meglitinide, in the treatment of type 2 diabetes mellitus. Arch Med Sci 2013; 9:936-43. doi: 10.5114/aoms.2013.34991.
19. Abiola D, Sathyapalan T, Hepburn D. Management of type 1 and type 2 diabetes requiring insulin. Prescriber 2016; 27:50-7.
20. Practical Insulin: A Handbook for Prescribing Providers, 4th ed. Virginia, USA: American Diabetes Association, Inc; 2015.
21. Sanlioglu AD, Altunbas HA, Balci MK, Griffith TS, Sanlioglu S. Clinical utility of insulin and insulin analogs. Islets 2013; 5:67-78. doi: 10.4161/isl.24590.
